# Genomes From Historic DNA Unveil Massive Hidden Extinction and Terminal Endangerment in a Tropical Asian Songbird Radiation

**DOI:** 10.1093/molbev/msac189

**Published:** 2022-09-06

**Authors:** Meng Yue Wu, Clara Jesse Lau, Elize Ying Xin Ng, Pratibha Baveja, Chyi Yin Gwee, Keren Sadanandan, Teuku Reza Ferasyi, Haminuddin, Rezky Ramadhan, Jochen K Menner, Frank E Rheindt

**Affiliations:** Department of Biological Sciences, National University of Singapore, Singapore, Singapore; Department of Biological Sciences, National University of Singapore, Singapore, Singapore; Department of Biological Sciences, National University of Singapore, Singapore, Singapore; Department of Biological Sciences, National University of Singapore, Singapore, Singapore; Department of Biological Sciences, National University of Singapore, Singapore, Singapore; Department of Biological Sciences, National University of Singapore, Singapore, Singapore; Faculty of Veterinary Medicine, Universitas Syiah Kuala, Darussalam-Banda Aceh, Indonesia; Faculty of Veterinary Medicine, Universitas Syiah Kuala, Darussalam-Banda Aceh, Indonesia; Faculty of Veterinary Medicine, Universitas Syiah Kuala, Darussalam-Banda Aceh, Indonesia; Prigen Conservation Breeding Ark, Prigen, Indonesia; Department of Biological Sciences, National University of Singapore, Singapore, Singapore

## Abstract

Quantifying the magnitude of the global extinction crisis is important but remains challenging, as many extinction events pass unnoticed owing to our limited taxonomic knowledge of the world’s organisms. The increasing rarity of many taxa renders comprehensive sampling difficult, further compounding the problem. Vertebrate lineages such as birds, which are thought to be taxonomically well understood, are therefore used as indicator groups for mapping and quantifying global extinction. To test whether extinction patterns are adequately gauged in well-studied groups, we implemented ancient-DNA protocols and retrieved whole genomes from the historic DNA of museum specimens in a widely known songbird radiation of shamas (genus *Copsychus*) that is assumed to be of least conservation concern. We uncovered cryptic diversity and an unexpected degree of hidden extinction and terminal endangerment. Our analyses reveal that >40% of the phylogenetic diversity of this radiation is already either extinct in the wild or nearly so, including the two genomically most distinct members of this group (*omissus* and *nigricauda*), which have so far flown under the conservation radar as they have previously been considered subspecies. Comparing the genomes of modern samples with those from roughly a century ago, we also found a significant decrease in genetic diversity and a concomitant increase in homozygosity affecting various taxa, including small-island endemics that are extinct in the wild as well as subspecies that remain widespread across the continental scale. Our application of modern genomic approaches demonstrates elevated levels of allelic and taxonomic diversity loss in a songbird clade that has not been listed as globally threatened, highlighting the importance of ongoing reassessments of extinction incidence even across well-studied animal groups.

***Key words*: extinction, introgression, white-rumped shama, conservation**.

## Introduction

The rate of global biodiversity loss has precipitously increased over the last few decades (Ceballos et al. 2015). This loss has been attributed to habitat degradation, introduction of invasive species, over-exploitation, climate change, and unsustainable and ill-regulated wildlife trade (Pimm et al. 2001; McNeely et al. 2009; Thomas et al. 2014; Eaton et al. 2015; Symes et al. 2018; Ng et al. 2021). Tropical regions, which are the center of global biological diversity, are at an especially high risk of unprecedented biodiversity loss (Jablonski et al. 2006).

Many studies have tried to quantify biodiversity loss using species as a currency to measure extinction rates (Balmford et al. 2003; Butchart et al. 2004; Ceballos et al. 2015; Cowie et al. 2017). While the definition of a species is a widely debated topic in biology (Wheeler and Meier 2000), its impact on biodiversity conservation is enormous in that species versus subspecies status can influence the amount of resources allocated to a taxon’s conservation (Isaac et al. 2004; Mace 2004; Morrison 2009; Stanton et al. 2019). Given that conservation resources are limited, an accurate picture of the distribution and phylogenetic distinctness of biodiversity is essential for a proper allocation of resources (Isaac et al. 2004; Stanton et al. 2019). Detailed taxonomic information is unavailable for most organismic groups on Earth, so large mammals and birds have widely served as the two most important indicator groups in such assessments, given the comparative wealth of biological data available for these taxa (Balmford et al. 2003; Butchart et al. 2004; Ceballos et al. 2015). Yet even these groups are not completely shielded from poor taxonomic resolution, leading to inaccurate evaluations of the volume and patterns of loss of evolutionary lineages. Egregious cases of hidden extinction events masked by inaccurate species delimitation or taxonomic uncertainty have been discovered even in birds (Cibois et al. 2009), casting doubt upon the sole reliance on species checklists in conservation. Consequently, an increasing number of biologists have come to advocate approaches which consider the genetic distinctness of lineages in conservation planning (Vane-Wright et al. 1991; Faith 1992; Jetz et al. 2014).

The members of the white-rumped shama *Copsychus malabaricus* complex constitute a songbird radiation that is assumed to be relatively well-inventoried in taxonomic terms, although species boundaries have been shifting somewhat among the 16 taxa widely recognized in this complex (including 15 traditional ones and one recently described to science, *ngae*; Wu and Rheindt 2022) (supplementary table S1, Supplementary Material online). They are widely known across Asia for their melodious songs and striking plumages, and range among the most popular cagebirds in the Southeast Asian and global bird trade (Nash 1993). Currently, the International Union for the Conservation of Nature (IUCN) classifies all recognized species of this complex as of “Least Concern” (BirdLife International 2021). However, member taxa in Southeast Asia are reported to be in rapid decline, with some populations on the brink of extinction or even extirpated due to a steep rise in trade and habitat loss over <2 decades (Eaton et al. 2015, 2016; Irham 2016; Rheindt et al. 2019; Wu and Rheindt 2022). The species complex is therefore listed among the top 12 priority groups for conservation by IUCN’s Asian Songbird Trade Specialist Group (Lee et al. 2016). With resources limited, a prioritization of distinct taxa is needed to maximize conservation success (Marris 2007), requiring information that is currently unavailable for this complex.

Previous phylogenetic work on the *C. malabaricus* complex has only included a subset of taxa, with full resolution still outstanding (Lim et al. 2010, 2017; Sangster et al. 2010; Chua et al. 2015; Wu and Rheindt 2022). Most prior work has relied on the signal of few DNA markers, chiefly restricted to the mitochondrion (Lim et al. 2010; Sangster et al. 2010; Chua et al. 2015), with all the associated susceptibility to artifacts of genetic introgression and single-marker phylogenetics (Ballard and Whitlock 2004; Hurst and Jiggins 2005; Rheindt et al. 2009; Rheindt and Edwards 2011). The only studies using genome-wide sequencing methods exhibited a restricted sampling regime limited to 3–9 out of 16 taxa (Lim et al. 2017; Wu and Rheindt 2022).

In this study, we used a combination of modern tissue samples and historic museum specimens with the goal of representing all taxa in the white-rumped shama *C. malabaricus* complex, especially rare insular ones that are seldom encountered in the wild now (supplementary table S2, Supplementary Material online). Whole genomes were generated using next-generation sequencing techniques to reconstruct the phylogeny and evolutionary trajectory of the complex, allowing us to identify conservation units. We then measured the evolutionary distinctness (ED) that each taxon contributes to the total evolutionary history of the complex to help prioritize conservation action (Jetz et al. 2014). We also conducted analyses into secondary gene flow among members of this radiation, revealing multiple incidents of introgression (Leathlobhair et al. 2018; Liu et al. 2019). Contrasting historic museum specimens collected in the late 1800s and early 1900s with modern tissue samples collected in the 2000s (supplementary table S2, Supplementary Material online), we demonstrate a pronounced decline of genomic diversity in shama populations over ∼100 years.

Our work points to considerable and unexpected loss of taxonomic and genetic diversity in what was thought to be a well-understood bird lineage. These results suggest that some of the effects of the current global extinction crisis may continue to be underestimated, and that urgent resources are necessary to bolster taxonomic work in the fight against diversity loss.

## Results

### Quality Filtering of Historic Museum Specimens

We prepared whole-genome libraries for a total of 60 individuals (supplementary table S2, Supplementary Material online) to generate genomes at a mean coverage of 8×, spanning 14 out of the 15 widely recognized taxa of the white-rumped shama complex following the taxonomy of Roberts et al. (2020) plus the newly described taxon *ngae* (Wu and Rheindt 2022), making for a total of 15 sampled taxa. The only missing form was the long-extinct taxon *mirabilis* from tiny Panaitan Island near Java, for which we were not able to source historic DNA material.

Out of the 60 individuals, a total of 22 were represented by DNA extracted from historic toepad material, covering 13 out of the 16 recognized taxa (supplementary table S2, Supplementary Material online). This sampling included two historic toepad samples of the Andaman shama *C. albiventris*, which is the only member of the complex that is simultaneously recognized as a species distinct from *C. malabaricus* in almost all modern taxonomies (supplementary table S1, Supplementary Material online).

Whole-genome resequencing is not a targeted technique and is capable of picking up exogenous contamination introduced to the specimen in the collection, with bacteria being the most likely contaminant. Thus, we used Fastq_screen to harvest reads that had mapped uniquely to the closely related Seychelles magpie-robin *C. sechellarum* genome (Wingett and Andrews 2018). We removed a total of seven samples, which had failed quality control due to a preponderance of unmappable sequence reads (“no hits” category, supplementary figs. S1 and S2, Supplementary Material online), indicative of highly degraded sequences. In addition, the two Andaman shama (*albiventris*) specimens exhibited high levels of *post mortem* damage in subsequent filtering steps and were also removed (supplementary fig. S3, Supplementary Material online).

In total, we successfully retained 59% (13 out of 22) of historic samples for genomic analysis after post-sequencing quality filtering, including from all taxa except *barbouri* and *albiventris.* Independent of the genome-wide sequence harvest, we recovered mitochondrial genomes for all 15 taxa sampled in this study, spanning all but four samples, the latter having to be removed for a high amount of “no hits” or low coverage (<3×).

### White-rumped Shama Phylogeny

**Fig. 1.**
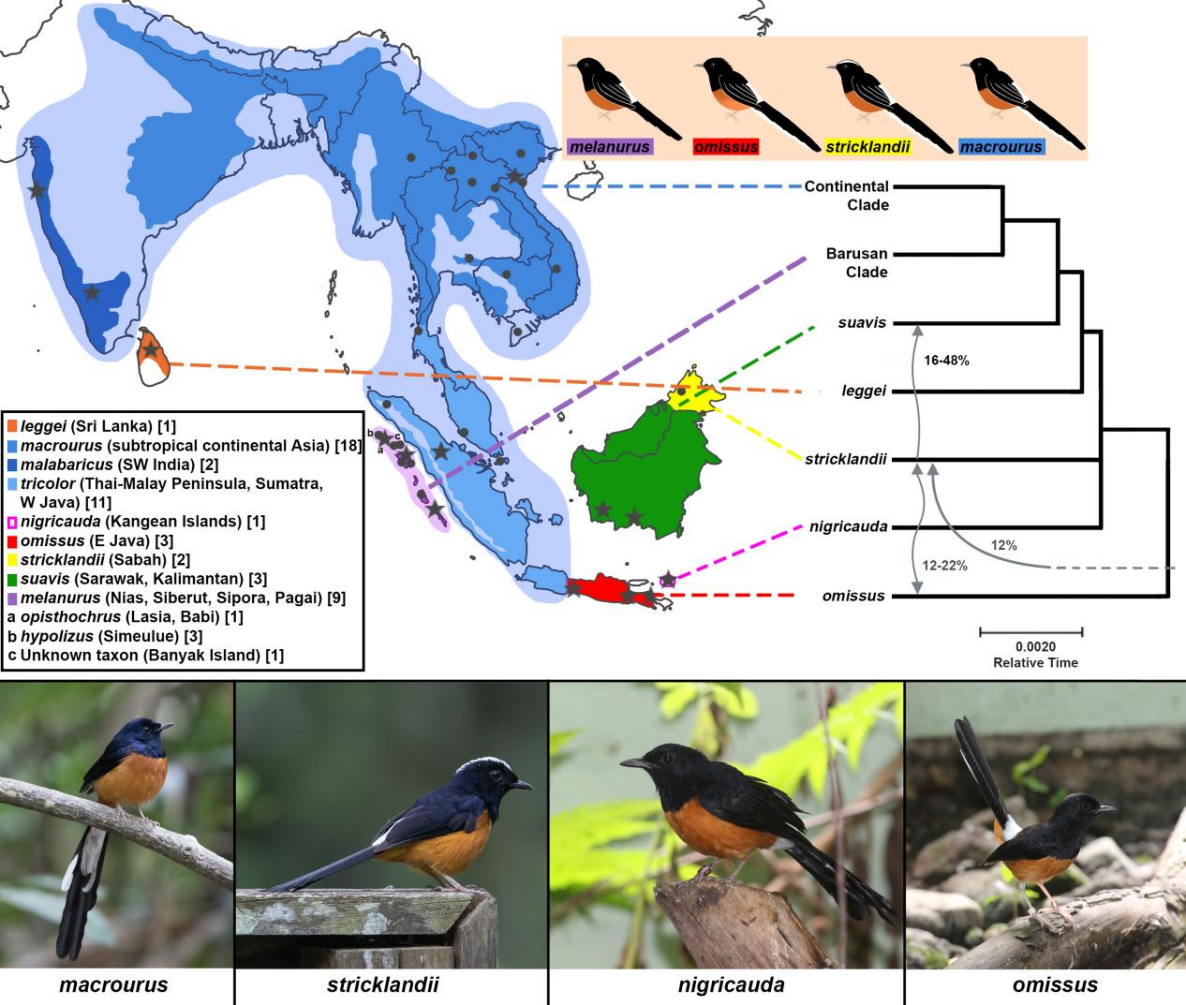
Phylogenetic analysis and distribution of the white-rumped shama *Copsychus malabaricus* complex. Distribution follows Roberts et al. (2020). Collection localities of DNA samples are given as black symbols, with circles and stars referring to fresh and historic samples, respectively. Taxa found to be paraphyletic or reciprocally embedded with each other are mapped together under the same color. Phylogenetic relationships are on the basis of 3,713 genome-wide sequence loci, each spanning 10 kb, from 51 whole-genome re-sequenced samples using maximum-likelihood RAxML at 100 bootstraps with all nodes having maximum branch support. The taxa *nigricauda* and *stricklandii* emerged as sister taxa with an extremely short branch leading to them. The resulting topology is shown as a chronogram using the RelTime-OLS algorithm (right) with oriental magpie-robin *C. saularis* as an outgroup (not shown). Arrows within the tree indicate the simplified patterns and magnitude of genetic introgression among tested taxa based on qpBrute analysis with 463,621 SNPs; for more detailed representations of allele sharing and gene flow, see supplementary fig. S7, Supplementary Material online. Continental Clade: *malabaricus*, *macrourus*, *tricolor*; Barusan Clade: *melanurus*, *opisthochrus*, *hypolizus*, *spp.* from Banyak Island. Sample size for each taxon given in square brackets. Photos at the bottom represent four distinct taxa within the complex. [Photos courtesy of Keita Y.C. Sin and Jochen K. Menner.]

Combining our historic DNA material with modern samples, we harvested 2,142,418 homologous single-nucleotide polymorphisms (SNPs) across all genomes. We concatenated all SNPs into one larger sequence and constructed a maximum likelihood topology using RAxML (Stamatakis 2014), calculating bootstrap values for each node (supplementary fig. S4, Supplementary Material online). We then harvested 3,713 genome-wide sequence loci, each spanning 10 kb, from a consensus fasta for each clade identified in the SNP tree and optimized for variability and low missing data to construct a highly supported maximum likelihood topology in RAxML (supplementary fig. S4, Supplementary Material online). This sequence-based RAxML topology was used to create a chronogram using RelTime-OLS (fig. 1) (Mello 2018). We also constructed two species trees using SNAPPER based on random subsets of 1,000 and 5,000 SNPs, respectively (Stoltz et al. 2021), given the great computational requirements for such analyses (supplementary fig. S4, Supplementary Material online).

We identified multiple monophyletic clades and novel topological arrangements (fig. 1). The central and eastern Javan taxa *javanus* and *omissus* clustered closely with each other, suggesting treatment as one Javan clade (labeled “*omissus*” in fig. 1 and supplementary fig. S4, Supplementary Material online). Similarly, close clustering was found in the shama populations and subspecies from the West Sumatran Islands (*melanurus*, *opisthochrus*, *hypolizus*, and unnamed populations; fig. 1), which were consequently grouped as one “Barusan” clade, named after the historic collective term for the West Sumatran islands as “Barusan Islands.” While there are multiple subspecies within each of these two clades, each subspecies generally emerged as monophyletic (supplementary fig. S4, Supplementary Material online). One individual (SIM45) obtained from a breeder on Simeulue Island, the home of *hypolizus*, was alleged to have been sourced from Nias Island, the home of *melanurus*. This individual emerged as genomically embedded within *hypolizus* (consistent with the island where its DNA was acquired) instead of *melanurus*, its claimed affinity (supplementary fig. S4, Supplementary Material online). Given the lack of direct historical, cultural or transportation connections between Simeulue and Nias, we consider this a case in which an erroneous provenance was inadvertently or deliberately attached to a traded bird, perhaps to increase its value.

The only shama grouping in which multiple taxa were found to be genomically paraphyletic and reciprocally embedded with one another was the continental clade comprising *macrourus*, *malabaricus* and *tricolor*, ranging across continental Asia, Sumatra, and western Java (blue in fig. 1 and supplementary fig. S4*a*, Supplementary Material online). The two Bornean taxa *stricklandii* and *suavis* emerged as polyphyletic with respect to each other (supplementary fig. S4, Supplementary Material online); however, this sequentially basal placement on the tree is likely due to introgression and allele sharing with two neighboring taxa (see “Presence of secondary gene flow” below). There is a slight difference in topology between the sequence-based and SNP-based trees, whereby *nigricauda* is sister to *stricklandii* in the former but more basal in the latter topology (supplementary fig. S4, Supplementary Material online). The eastern Javan clade emerged as basal to the entire complex (fig. 1). While our species trees, i.e., those constructed with methodologies based on the multi-species coalescent, were generally characterized by low branch support, with ∼55% of branches collapsed, none of the well-supported branches contradicted our maximum likelihood trees even in the presence of secondary gene flow (supplementary fig. S4, Supplementary Material online).

**Fig. 2.**
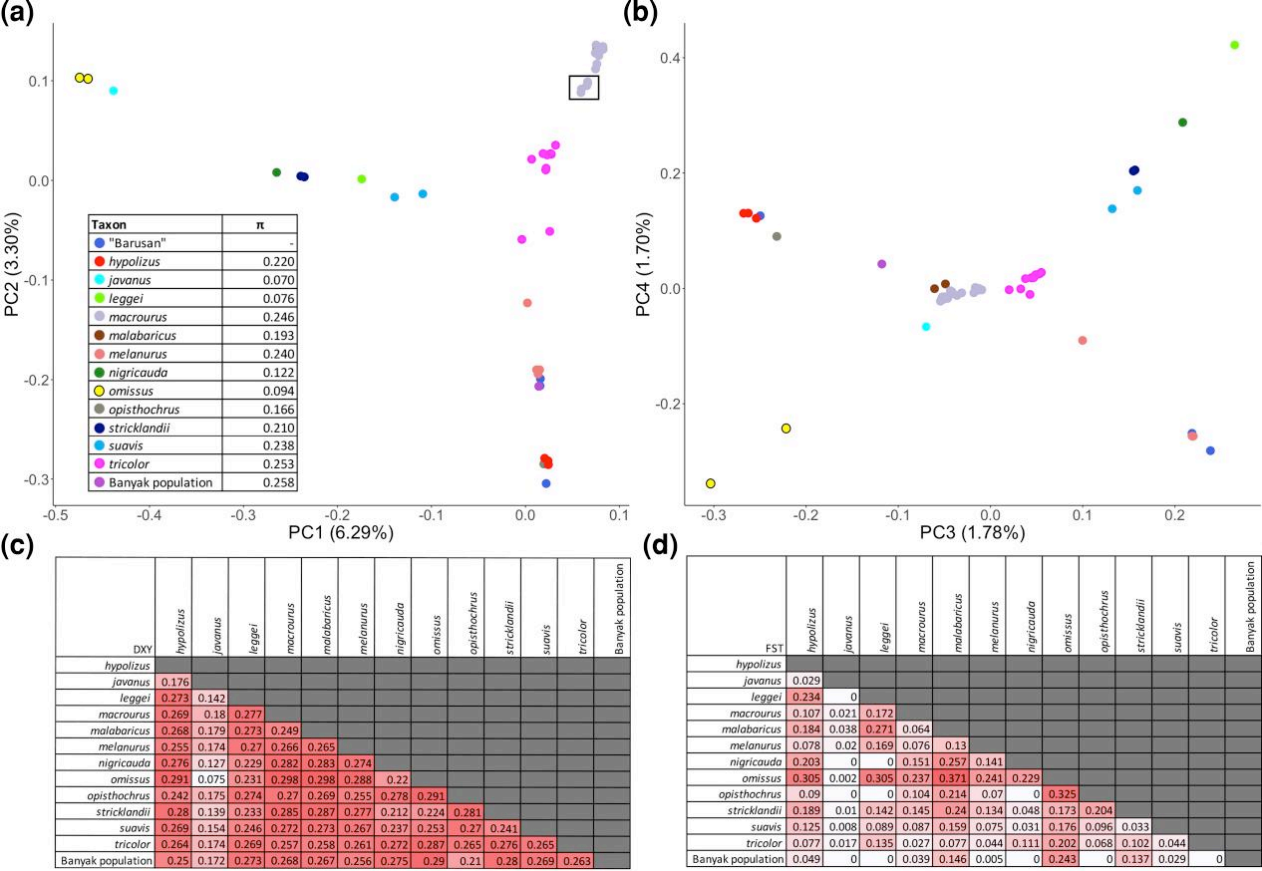
Summary of genomic diversity within and between all commonly recognized taxa of the white-rumped shama *C. malabaricus* complex. PCA of 51 shama samples based on 2,579,997 SNPs using PCAngsd for four principal components (PC) with (*a*) PC1 and PC2, and (*b*) PC3 and PC4. The *macrourus* samples from the northern part of the taxon’s range are shown within a black box. The points labeled “Barusan” (dark blue) refer to museum samples collected from the West Sumatran islands for which exact locality information was not available. The global nucleotide diversity (*π*) values for each taxon are given in the table inside panel (*a*). The percentage of total variation explained by each PC is shown in brackets. Global divergence values expressed in terms of (*c*) *F*_ST_ and (*d*) *D*_XY_ are given for each pairwise taxon comparison.

Principal component analysis (PCA) based on 2,579,997 SNPs was consistent with our phylogenetic analyses, yielding the deepest division between the Javan individuals (*omissus*) and the remainder of the complex (fig. 2*a*), with other taxon clusters emerging as distinct at lower PCs (fig. 2*b*). Conversely, admixture analysis using NGSadmix, which was carried out from *K* = 2 to *K* = 15 (supplementary fig. S5, Supplementary Material online), vacillated between reflecting the signal of primary phylogenetic relationships versus secondary gene flow or introgression (see below), depending on how many clusters were inferred. The taxon *omissus* emerged as highly differentiated from the other taxa in terms of *D*_XY_ and *F*_ST_ summary statistics (fig. 2*c*and*d*), although the picture was less clear for *F*_ST_, presumably because the latter is sensitive to artifacts of differing sample sizes per taxon. The genomic nucleotide diversity of some insular taxa, especially *omissus*, *leggei*, and *javanus*, turned out to be only roughly half that of the nucleotide diversity of most other taxa (fig. 2*a*).

Two individuals (JF1793 and JF1795) from Vietnam were consistently distinct from other *macrourus* samples in NGSadmix at *K* ≥ 8 (shown with asterisks in supplementary fig. S5, Supplementary Material online). These individuals do cluster slightly apart from the main *macrourus* cluster in PCA (black box in fig. 2*a*), together with an individual from Cambodia (JF318) and two samples from Vietnam with unknown localities (NRM20036774 and NRM20036776). This clustering pattern is supported by the SNP-based tree, in which the aforementioned group of five individuals is sister to the remainder of *macrourus* samples (supplementary fig. S4*a*, Supplementary Material online). However, the latter three samples among these five (JF318, NRM20036774, and NRM20036776) did not possess the same genomic signature as JF1793 and JF1795 in NGSadmix analysis (supplementary fig. S5, Supplementary Material online). Further inspection revealed these latter three individuals were collected in southern Indochina, while JF1793 and JF1795 are from the north of Indochina, supporting the notion of clinal latitudinal population differentiation within *macrourus*, at least in the main Indochinese portion if its ranges.

The museum samples sourced from the Naturalis Biodiversity Center, Leiden, were often associated with brief or illegible locality information; while we can be certain that they are from the Barusan islands, we often do not know which island they were collected on (supplementary table S2, Supplementary Material online). Our analyses allowed us to infer the most likely islands these samples are from based on their clustering in our tree, PCA, and admixture results (supplementary table S2 and figs. S4 and S5, Supplementary Material online).

### Mitogenomic Divergences

A cytochrome-*c* oxidase subunit 1 (COI) mitochondrial divergence threshold of 2–3% has been postulated in the literature to provide corroboration for species-level recognition of closely related bird species (Hebert et al. 2004; Kerr et al. 2007). The deepest pairwise mitochondrial divergences (as compared with the next most closely related clade) were—unsurprisingly—associated with the two taxa that emerged as most basal on the genome-wide RAxML tree, namely the eastern Javan clade (average COI pairwise distance: 2.59%; mitogenome: 2.75%; cyt-*b*: 3.80%) and the taxon *nigricauda* from Kangean Island (COI: 2.70%; mitogenome: 2.39%; cyt-*b*: 3.29%) (supplementary tables S3–S5, Supplementary Material online). Mitochondrial pairwise divergences remained around the COI barcoding threshold for the next most basal taxa on the genome-wide RAxML tree, i.e., *stricklandii* from Sabah (COI: 2.57%; mitogenome: 2.23%; cyt-*b*: 2.67%), *leggei* from Sri Lanka (COI: 2.83%; mitogenome: 2.54%; cyt-*b*: 3.08%), and *suavis* from Sarawak (COI: 2.48%; mitogenome: 2.09%; cyt-*b*: 2.96%) (supplementary tables S3–S5, Supplementary Material online). Inter-taxon pairwise mitogenomic differentiation involving *barbouri* from Maratua Island (COI: 1.16%; mitogenome: 1.51%; cyt-*b*: 2.57%), the main continental clade (COI: 1.93%; mitogenome: 1.48%; cyt-*b*: 2.32%), and the Barusan clade (COI: 1.69%; mitogenome: 1.65%; cyt-*b*: 2.09%) dropped below 2% (supplementary tables S3–S5, Supplementary Material online).

We used the Andaman shama *C. albiventris* as a yardstick for mitochondrial divergence thresholds in this complex because it has consistently been classified as an independent species by the vast majority of historic and modern taxonomic treatises (supplementary table S1, Supplementary Material online). The Andaman shama was diverged by 3–4% from all other individuals (COI: 3–4%; mitogenome: 3–4%; cyt-*b*: 3–7%; supplementary tables S3–S5, Supplementary Material online) with one exception: the newly described taxon *ngae* (Wu and Rheindt 2022), sampled from Langkawi Island, had a mitochondrial haplotype that was only around 1% (COI: 1.01%; mitogenome: 1.2%; cyt-*b*: 1.2%) diverged from the Andaman shama and fell outside the main continental clade of white-rumped shamas (supplementary fig. S6, Supplementary Material online). Unfortunately, both the Langkawi and Andaman samples were derived from particularly degraded museum material and did not pass quality control for whole-genome analysis, leaving their placement on the genomic phylogeny undetermined.

The mitogenomic tree topology exhibited incongruences with our genomic RAxML tree (supplementary fig. S6, Supplementary Material online), consistent with the known limitations of phylogenetic approaches exclusively relying on mtDNA (Ballard and Whitlock 2004; Hurst and Jiggins 2005; Rheindt and Edwards 2011). Topological disagreements notwithstanding, the mitogenomes also reflected the eastern Javan clade as basal to all other members of the complex (supplementary fig. S6, Supplementary Material online).

### Presence of Secondary Gene Flow

The two Bornean taxa, *stricklandii* from Sabah and *suavis* from the remainder of Borneo, abut in a narrow geographical hybrid zone, with reports of low levels of hybridization (Lim et al. 2017). This distribution pattern and the polyphyletic placement of these taxa in the genome-based phylogeny (supplementary fig. S4, Supplementary Material online) are consistent with a scenario of secondary gene flow. We assessed the presence of secondary gene flow between these two taxa in pairwise comparison, and between them and other taxa that could have come into contact with them, both presently and ∼15,000 years ago when land bridges between Borneo and surrounding landmasses last existed (Sarr et al. 2019). As such, we chose individuals from the continental Asian clade, eastern Javan clade, *nigricauda* from Kangean and the two Bornean taxa *suavis* and *stricklandii* to investigate potential secondary gene flow events using admixture graph analysis in qpBrute (Leathlobhair et al. 2018; Liu et al. 2019).

We detected six optimal models, which showed only negligible differences in *K* values and all converged on one similar gene flow scenario, albeit with slight differences in the exact proportions of gene flow (fig. 1, right panel). The resulting admixture graph indicates gene flow from *stricklandii* into *suavis* at a genomic contribution of 16–48% depending on the model (fig. 1). There is also secondary allele sharing between *stricklandii* and the eastern Javan clade at a proportion of 12–22% (fig. 1). We additionally detected a 12% contribution into *stricklandii* from an unknown donor (fig. 1), possibly referring to an extinct lineage. The high number of secondary gene flow events centered around *stricklandii* suggests that this taxon seems to have one of the most complex demographic histories among white-rumped shamas.

Some of the six models incorporated “ghost” lineages, which could be a modeling artifact or may constitute extinct lineages that are closely related to direct ancestors of extant populations (supplementary fig. S7, Supplementary Material online). We interpreted the allelic contributions from any given “ghost” lineage to be from the extant population it is most similar to (fig. 1), but also allowed for their interpretation as originating from unsampled and possibly extinct populations (supplementary fig. S7, Supplementary Material online).

### Evolutionary Distinctness of Members of the Complex

We measured ED, the total phylogenetic diversity (PD) in the complex, and the “evolutionarily distinct and globally endangered score” (EDGE) following Jetz et al. (2014). In our calculations, ED—and thus the loss in PD—generally increases as a clade becomes more basal in the phylogeny (table 1). When combining distinctness and extinction risk using EDGE, the Barusan clade, *nigricauda* from Kangean, and the eastern Javan clade containing *omissus* and *javanus* emerged with the highest scores (table 1). The EDGE score for *nigricauda* is probably an underestimate as we used the weightage for “Critically Endangered” even though it is most likely extinct in the wild, which would suggest a higher weightage and thus a higher EDGE score. Loss in PD is unaffected by conservation status and is here used for more accurate comparisons.

**Table 1.** Measurements of Various Proxies of Distinctness and Conservation Status.

Taxon/Clade	Conservation Status	ED	Total PD	EDGE	Loss in Phylogenetic Diversity
Continental clade	LC	0.00604	0.0448	0.00602	13.5
Barusan clade	CR	0.00604		2.78	13.5
*suavis*	LC	0.00659		0.00656	14.7
*leggei*	LC	0.00687		0.00685	15.3
*stricklandii*	LC	0.00606		0.00604	13.5
*nigricauda*	CR^a^	0.00606		2.78	13.5
*omissus*	CR	0.00716		2.78	16.0

Note.—Evolutionary distinctness (ED), total phylogenetic diversity of the complex (PD), and “evolutionarily distinct and globally endangered” (EDGE) scores within the white-rumped shama *C. malabaricus* complex are calculated from RelTime-OLS branch lengths following Jetz et al. (2014). Loss in phylogenetic diversity per taxon/clade is calculated as ED/PD × 100. Conservation status reflects status assessments by the authors based on the criteria set out by the IUCN; the IUCN itself has not issued any status assessments for the lineages here listed because it uses traditional taxonomic arrangements. Conservation status abbreviations: CR, Critically Endangered; LC, least concern. “Continental clade” refers to *malabaricus*, *macrourus*, and *tricolor* centered in mainland Asia; Barusan clade refers to all taxa on the Barusan Islands.

a Refer to taxa that are putatively extinct in the wild. Values are rounded to three significant figures.

### Changes in Genetic Diversity Over Time

**Fig. 3.**
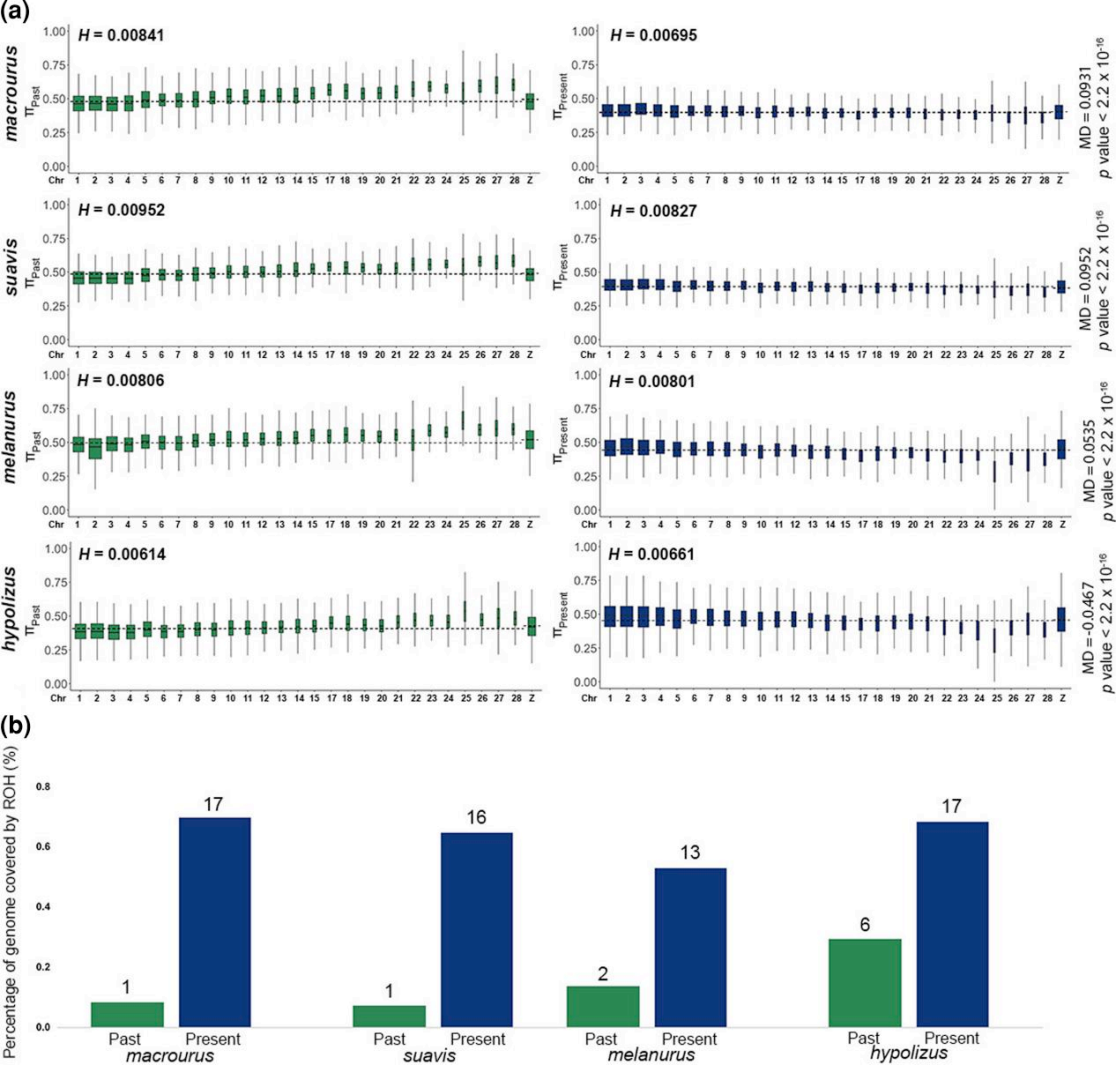
Comparison of population parameters between two time points across four white-rumped shama taxa—*macrourus*, *suavis*, *melanurus*, *hypolizus*. (*a*) Chromosome-wise boxplots of nucleotide diversity, as measured in 50 kb sliding windows with a step size of 10 kb across the genome for samples collected one century ago (left, green) and modern samples (right, blue). Only major chromosomes are shown along *x*-axis, for additional chromosomes see supplementary fig. S8, Supplementary Material online. The horizontal black dotted lines denote the mean nucleotide diversity of autosomes and the Z chromosome, which is generally higher in samples collected one century ago than in modern samples. Global heterozygosity (*H*) values given above each sample. (*b*) Summation of all runs of homozygosity (ROH) in each population as a percentage of the genome, provided for the samples collected one century ago (past) and modern samples (present). The number of ROHs found in each population is given as an integer above each bar.

We measured nucleotide diversity and heterozygosity for four taxa (*macrourus*, *suavis*, *melanurus*, *hypolizus*) for which genome sequences from two time points approximately a century apart were available, to determine the change in diversity over the last 100 years. Many measures of genetic diversity are subject to sample size bias. In order to rule out potential biases, we measured nucleotide diversity and heterozygosity using representative male samples from each taxon and time point with two exceptions—the modern samples for *macrourus* and *suavis*. The sex of these two samples is likely male, but remains unknown and could potentially be female, even though the genetic diversity values of their Z chromosome do not suggest so (fig. 3 and supplementary fig. S8, Supplementary Material online).

We generally detected a significant decrease in nucleotide diversity across the genome, with the Z chromosome experiencing a larger decrease compared with the autosomes, in all taxa except *hypolizus* (fig. 3*a* and supplementary fig. S8, Supplementary Material online). The heterozygosity values follow a similar pattern (fig. 3*a*). Comparing these values with genome-wide heterozygosity values of other birds, the four shama taxa fare slightly better than other endangered species (supplementary tables S6 and 7, Supplementary Material online). We found that runs of homozygosity (ROH) have substantially increased across all these four taxa over ∼100 years, now covering a considerably larger proportion of the genome than they did a century ago (fig. 3*b* and supplementary table S8, Supplementary Material online). When measures of differentiation (*D*_XY_ and *F*_ST_) between historic and modern samples were plotted across the genome, *hypolizus* displayed a particularly pronounced variance as compared with the other three taxa (Turner et al. 2005; Harr 2006) (supplementary fig. S9, Supplementary Material online).

## Discussion

Earth’s biodiversity has been subject to significant anthropogenic environmental impacts in the Anthropocene (roughly the period since the world wars), with an increasing recognition of the importance of biodiversity conservation (Rands et al. 2010; Mittermeier et al. 2011). On a practical level, a great proportion of today’s conservation management uses species as a currency, for example when quantifying diversity or when designing conservation measures tailored to specific endangered species (Isaac et al. 2004; Mace 2004; Morrison et al. 2009; Stanton et al. 2019). At the same time, there is increasing awareness that taxonomic uncertainty and a lack of biological knowledge create serious limitations for the species-based conservation approach, even in well-known organismic groups. Next-generation sequencing and the rise of relevant bioinformatic pipelines have created new opportunities, allowing for great strides to be made to unravel the hidden diversity in birds (Zhang et al. 2019; Gwee et al. 2020; Baveja et al. 2021; Ng et al. 2021), mammals (Mittermeier et al. 2008; Trigo et al. 2013; Fennessy et al. 2016), and reptiles (Leaché and Fujita 2010; Fujita and Papenfuss 2011; Wagner et al. 2014). Here, we shed light on the evolutionary relationships in shama songbirds of the *C. malabaricus* complex, with significant ramifications for their conservation.

### Evolutionary History of White-rumped Shamas

Past attempts to unravel the evolutionary trajectory of the *C. malabaricus* complex have been limited to few markers whose signal was dominated by mitochondrial DNA (Lim et al. 2010; Chua et al. 2015) or compounded by incomplete taxon sampling (Lim et al. 2010; Chua et al. 2015; Lim et al. 2017; Wu and Rheindt 2022). Here, we used more than 37,130,000 bp from throughout the whole genomes of 13 currently recognized taxa and 51 individuals to shed light on the evolutionary history of white-rumped shamas (Roberts et al. 2020). Our phylogeny indicated the existence of seven major clades within the complex (fig. 1 and supplementary fig. S4*a*and*b*, Supplementary Material online). Surprisingly, the genomically most distinct member of the complex is a clade of two eastern Javan taxa (*omissus* and *javanus*) on the brink of extinction, which have never previously been considered to be a distinct species (fig. 1). The second most distinct member is a taxon from the small islands of Kangean (*nigricauda*) now widely believed to be extinct in the wild. Our phylogeny also confirmed the distinctness of *stricklandii* from Sabah (fig. 1), which is variably recognized at the species or subspecies level (supplementary table S1, Supplementary Material online). Some taxa whose DNA emerged as too degraded for inclusion in genomic analyses still yielded mitogenomic DNA of sufficient quality. For one of them, taxon *barbouri* from Maratua Island, the mitogenomic phylogeny (supplementary fig. S6, Supplementary Material online) confirmed its fairly diverged position as previously demonstrated by Chua et al. (2015) while the distinctness of the other, *albiventris* from the Andamans, was also corroborated by mtDNA. The slight discrepancies regarding the placement of *stricklandii* between the sequence-based and SNP-based genomic trees were most likely an artifact of gene flow between *suavis* and *stricklandii* (fig. 1).

The detection of the eastern Javan clade (*omissus* and *javanus*) as most basal within white-rumped shamas calls for immediate conservation action, as these two taxa had previously passed for weak subspecies and gone under the conservation radar. Meanwhile, the unique reproductive display and behavior of these eastern Javan shamas is well known in breeders’ and traders’ circles and is partly responsible for their great endangerment (JKM, personal observation). A high ED of Javan members among Sundaic radiations is not entirely unique to *Copsychus*: examples of Javan taxa of high genetic or bioacoustic distinctness within their Southeast Asian species complexes include *Mixornis* babblers (Cros and Rheindt 2017) and *Alophoixus* bulbuls (Fuchs et al. 2015).

### Secondary Gene Flow Dynamics Across Southeast Asia

Secondary gene flow among species is commonplace in nature, and is especially prevalent in songbirds (Rheindt and Edwards 2011; Toews et al. 2016; Stryjewski and Sorenson 2017; Gwee et al. 2020; Manthey et al. 2020). We performed gene flow analyses using a modern extension of the ABBA–BABA approach (Green et al. 2010; Patterson et al. 2012) to investigate potential allelic exchanges among lineages within the complex. The taxa *suavis* and *stricklandii* share the island of Borneo, their ranges abutting along a narrow and well-characterized hybrid zone (Lim et al. 2010, 2017). Unsurprisingly our analyses detected evidence of secondary gene flow between *suavis* and *stricklandii* (fig. 1), corroborating the results of past studies (Lim et al. 2017).

But the signal of secondary gene flow was not restricted to lineages on the same islands, attesting to the Sundaic region’s complex history of fluctuating sea levels and exposed land bridges providing conduits of gene flow among landmasses (Cros et al. 2020). A succession of glacial cycles has allowed for sea level recessions during ice ages to expose continental Sundaland in currently submerged areas between present-day Java and Borneo. This may have allowed presently isolated lineages to interact and admix (Cros et al. 2020). We detected secondary gene flow between *stricklandii* from Sabah and the eastern Javan clade (fig. 1), even though they are distantly related in the complex, adding to previous insights that secondary gene flow does not necessarily have to be restricted to sister lineages (Dasmahapatra et al. 2012; Jacobsen and Omland 2012; Edelman et al. 2019; Gwee et al. 2020).

We also detected a genomic contribution into the taxon *stricklandii* from an unsampled or ancestral lineage (fig. 1). Such patterns are an increasingly common feature of modern gene flow analyses and likely reflect contributions from now-extinct lineages (Zhang et al. 2019). However, determining the exact identity of this genomic contribution goes beyond the scope of this study.

There is also extensive gene flow between the three taxa within the “Continental” clade (supplementary fig. S5, Supplementary Material online), leading to the low mitochondrial divergences and the paraphyletic placements of the three taxa (*malabaricus*, *macrourus*, *tricolor*).

### Extinction and Endangerment Have Been Considerably Underestimated in Shamas

Because of our extensive knowledge about their biology and taxonomy, birds are a widely-used model organism and indicator group in conservation. For instance, Endemic Bird Areas (EBAs) are used as general indicators of organismic endemism by conservation planners to focus efforts on areas with concentrations of unique species (Stattersfield et al. 1998). Our genomic comparisons among white-rumped shamas, however, challenge the assumption that all birds are well understood in their conservation needs, and pinpoint egregious instances of hidden extinctions or species in stages of terminal endangerment without any formal recognition. Given that these shamas have been thought to be exceptionally well understood and have not been flagged by the IUCN as threatened, it comes as a surprise that 43% of the PD of this complex was mapped as extinct or terminally endangered (i.e., taxa or clades designated CR in table 1).

Members of the white-rumped shama *C. malabaricus* complex are among the most widely kept and intensely traded birds in Asia. Despite their great endangerment and local extinction in certain regions, such as Indonesia and Malaysia, they have not been recognized as threatened by the IUCN Red List (BirdLife International 2021) largely on account of the fact that most populations have traditionally been united into one wide-ranging species (*C. malabaricus*). Using genomic data and accounting for secondary gene flow across the complex archipelagic landscape of Southeast Asia, we show that the genomically most distinct unit within the complex—which has hitherto never been recognized at the species level—is close to extinction in the wild, and that 2–3 other deeply divergent lineages that can plausibly be recognized at the species level are already extinct in the wild (see supplementary material online). Crucially, all these extinct or terminally endangered units have hitherto passed for subspecies or sometimes even been synonymized in the world’s major avian checklists (supplementary table S1, Supplementary Material online). This tally attests to a considerable incidence of hidden endangerment and extinction in an avian lineage previously thought to be exceptionally well understood.

The results of this study advocate a global drive in increased taxonomic research, especially when supplemented by novel genomic data and analytical approaches. Only a concerted effort among the world’s biologists can lead to a more realistic appraisal of the taxonomic diversity on the planet, which is a prerequisite in any efforts to safeguard the Earth’s biological diversity.

### Extinction and Endangerment Process Driving Changes in Shama Genomes

When comparing genomes between modern individuals and those collected roughly a century ago across four taxa—*macrourus*, *suavis*, *hypolizus*, and *melanurus—*we detected a 2–8-fold increase in the length and number of ROHs over time, indicating that the genomes of these taxa are becoming more homogeneous with time (fig. 3*b* and supplementary table S8, Supplementary Material online). This rising genomic impoverishment is supported by a decrease in overall nucleotide diversity and heterozygosity in *macrourus*, *suavis*, and *melanurus* (fig. 3) and is consistent with a series of major and minor bottleneck events and population declines that would have eroded allelic variability over ∼100 years. This worrying trend is likely to translate into an overall decrease in effective population size over the years given the correlation between the latter and nucleotide diversity.

Only *hypolizus*, a taxon endemic to the West Sumatran island of Simeulue, exhibited an increase rather than decrease in nucleotide diversity and heterozygosity (fig. 3*a*), accompanied by a 2-fold increase in the percentage of ROHs across the genome (rather than a threefold to 10-fold increase in the other three taxa; fig. 3*b*). On the surface, this result would suggest a gain in population-genetic diversity in *hypolizus*, yet the present-day field situation points to a different scenario: *hypolizus* shamas probably went extinct in the wild around 2015 (Rheindt et al. 2019), and our modern *hypolizus* sample (SIM44) comes from a local trader from Simeulue who breeds multiple shama taxa. The inflated nucleotide diversity value is likely to be an artifact of cross-breeding and the infiltration of alien alleles from the mainland clade.

Compared with heterozygosity estimates of other birds sourced from the literature, the modern values of these four shamas are generally higher than those of threatened species elsewhere (supplementary tables S6 and S7, Supplementary Material online). However, when projecting the decline over the last century forwards, action needs to be taken now to avoid an unsustainable impoverishment in genetic diversity in these shamas.

### Extinction-in-progress of the White-rumped Shama Radiation

The current conservation status of white-rumped shamas belies the results of our genomic analyses, which have uncovered cryptic diversity and paint a bleak picture of hidden extinction events and instances of terminal endangerment. Multiple clades within the white-rumped shama are being threatened by unsustainable wildlife trade and habitat degradation (Chng and Eaton 2016; Irham 2016; Rheindt et al. 2019).

The taxon *nigricauda* from the Kangean Islands, which emerged as the second most distinct taxon of the radiation (fig. 1) and comprises ∼14% of the PD of genomically sampled taxa within the complex, is widely assumed to be extinct in the wild now owing to unsustainable trapping (Eaton et al. 2015; Irham 2016). Another cumulative ∼30% of the radiation is terminally endangered, comprising a clade made up of the two most easterly Javan taxa (*javanus* and *omissus*) and the Barusan clade (table 1). This genome-based assessment does not even include another three phylogenetically distinct insular taxa for which we only managed to obtain mitogenomic DNA (Andaman *albiventris*, Maratua *barbouri*, and *ngae* from Langkawi Island), the latter two of which are either extinct in the wild or nearly so (see Supplementary material online).

The eastern Javan clade’s surprising placement as the genomically most basal lineage accounts for 16% of the total PD of the complex (table 1). This distinct genomic make-up likely encompasses a multitude of unique alleles, adaptations, and functions that set eastern Javan individuals apart from other members of the complex, consistent with the unusual behavioral innovations documented from this population (see Supplementary material online). While its exact fate in the wild has not received much attention, it is the most highly prized population in Indonesian breeders’ circles and is heavily poached, with the only surviving population currently known from one national park in southeast Java (JKM, personal observation). The imminent extinction in the wild of this clade would represent a dangerous step toward total extinction as Javan birdkeepers are known to hybridize endangered native birds in captivity with closely related species for lack of new recruitment (Sadanandan et al. 2020; Baveja et al. 2021).

Although less phylogenetically distinct, the Barusan clade consists of three named subspecies and multiple unnamed island populations, only one of which was thought to survive on Siberut Island in 2018 in single or low double digits, although captive individuals continue to exist for two other island populations (Rheindt et al. 2019).

The extinction threat may progressively encompass the entire complex in the long run, with *macrourus* and *suavis*, both commonly thought to be safe in the wild, experiencing rising genomic homogenization, with a significant decrease in nucleotide diversity and increase in the length and number of ROHs over the course of a century (fig. 3). Prompt and urgent conservation action is required to halt a further progression of extinction in white-rumped shamas. Any further delays in ex situ or in situ conservation measures may jeopardize the survival of a significant portion of genetic diversity in this complex.

### A Conservation Blueprint Based on a New Classification

To facilitate urgent conservation action, we present a new taxonomic classification based on our genomic data supplemented by insights from morphology and reproductive behaviour (see Supplementary material online). Our new classification lists two species-level lineages and one distinct subspecies that are widely assumed to be extinct or extinct in the wild (*nigricauda*, *barbouri*, and *ngae*, respectively), followed by one species-level lineage that is terminally endangered (*omissus*), as well as additional species-level lineages of concern (e.g., *malabaricus*, *stricklandii*).

Conservation in many countries, especially in Southeast Asia, is often constrained by taxonomic bias, affording attention only to species-level lineages. Imperfect or erroneous taxonomic classifications will often lead to endangered species being ignored or left out of conservation planning (Sadanandan et al. 2020; Baveja et al. 2021; Ng et al. 2021). It is hoped that this new classification of shamas will help conservationists adjust action to lineages that have previously been overlooked.

## Methods and Materials

### Taxon Sampling

We obtained a total of 22 historic toepad samples and 34 modern tissue samples from various museums and through fieldwork conducted in the Indonesian archipelago (supplementary table S2, Supplementary Material online). We sampled all taxa of the *C. malabaricus* complex following the taxonomy of Roberts et al. (2020), with the exception of the long-extinct *mirabilis*.

### Laboratory Procedures and Sequencing

The DNA of modern tissue samples was extracted using the DNeasy Blood and Tissue Kit (Qiagen, Hilden, Germany) following the manufacturer’s protocol with an additional RNase treatment applied. Toepad samples from dried historic skin specimens were extracted for DNA under sterile conditions inside a dedicated ancient-DNA facility using the same kit with slight modifications as previously documented (Chattopadhyay et al. 2019; Wu et al., 2020).

Fresh modern DNA was sheared into a targeted size of 250 bp using a Bioruptor Pico sonication device (Diagenode, Marlborough, MA, USA). Whole-genome libraries were prepared using NEBNext Ultra DNA Library Prep Kit for Illumina (New England Biolabs, Ipswich, MA, USA) for both fresh and historic samples, with additional modifications for the latter following Chattopadhyay et al. (2019) and Wu et al. (2020).

Multiple extraction and library negatives were included to detect contamination. DNA concentrations were ascertained using a Qubit 2.0 high sensitivity DNA Assay kit (Invitrogen, Waltham, MA, USA), and fragment sizes were assessed using an AATI Fragment Analyzer (Agilent, Santa Clara, CA, USA). The negatives were also quantified using a Qubit 2.0 assay and AATI to ensure absence of contaminating DNA. Once checked, libraries were sequenced at NovogeneAIT Genomics (Singapore) on the Illumina Hiseq 4000 platform to produce 150-bp paired-end reads.

### Initial Filtering and SNP Calling

Across the 56 samples, we removed adaptor sequences with cutadapt (Martin 2011) and aligned reads to the Seychelles magpie-robin *C. sechellarum* assembly (GenBank accession: GCA_013398635.1) using BWA-MEM (Li 2013). Low-quality reads (MAPQ score < 20) were filtered with SAMtools v1.6-1 (Li et al. 2009) to ensure unique mapping. Picard v2.17.3 (http://broadinstitute.github.io/picard/) was subsequently used to assign read group information and mark duplicates. Lastly, we used RealignedTargetCreator and IndelRealigner as implemented in the Genome Analysis Toolkit (GATK) v3.8-0 (Broad Institute, USA) (McKenna et al. 2010) to realign and refine the original alignment. The output bam format files were checked in Qualimap v2.2.1 (Okonechnikov et al. 2016) for mapping quality and sequencing bias before variant calling (supplementary table S9, Supplementary Material online).

After adaptor removal, we excluded sequences that are potentially representative of exogenous contamination in our historic samples by mapping our reads to four reference genomes—(i) human (GenBank Assembly Accession: GCA_000001405.28), (ii) Seychelles magpie-robin (GenBank accession: GCA_013398635.1), (iii) a compilation of all available bacterial genomes on the RefSeq database, and (iv) an *Aerodramus* swiftlet genome (unpublished data) because swiftlets were sequenced on the same Illumina lane. Using Fastq_screen (Wingett and Andrews 2018), we extracted reads that mapped uniquely to the Seychelles magpie-robin genome before re-entry into the pipeline. This removal of reads resulted in the exclusion of seven samples, including the sole representative of the taxon *barbouri*.

Historic museum samples are known to experience severe *post mortem* DNA damage that can confound downstream analysis, necessitating the application of sophisticated analytical tools (Pääbo et al. 2004; Billerman and Walsh 2019). We used mapDamage 2.0 to visualize damage patterns, and removed an additional two samples that showed elevated substitution rates indicative of excessive DNA damage. We also rescaled the quality scores of the remaining historic samples using a Bayesian statistical model of DNA damage as implemented in mapDamage 2.0 (Jónsson et al. 2013).

### Phylogenomic Analysis

We retained a total of 52 high quality samples for genomic variant calling using ANGSD with a strict filter regime, including the Oriental magpie-robin as an outgroup (-uniqueOnly 1, -remove_bads 1, -only_proper_pairs 1, -SNP_pval 1e-6, -trim 5, -minMapQ 30, -minQ 30, -minMaf 0.05, -minIndDepth 3, -geno_mindepth 3, 90% presence across individuals) (Korneliussen et al. 2014). SNPs with a pairwise linkage disequilibrium correlation coefficient higher than 0.5 as measured in PLINK v1.90 using a window size of 25 and a step size of 10 were removed (Purcell et al. 2007). We also removed invariant sites using acsbias.py (https://github.com/btmartin721/raxml_ascbias) before tree search. We created a maximum likelihood tree with the resulting dataset comprising 2,142,418 concatenated SNPs using the ASC_GTRGAMMA model in raxmlHPC-PTHREADS v 8.2.12 for 100 bootstraps with an oriental magpie-robin *C. saularis* as an outgroup (Stamatakis 2014).

Consensus fasta sequences were called using ANGSD -doFasta for each of the seven major phylogenetic groups in our genomic SNP tree—(i) East Java (*omissus*), (ii) Kangean Island (*nigricauda*), (iii) Sabah (*stricklandii*), (iv) remaining Borneo (*suavis*), (v) Sri Lanka (*leggei*), (vi) Barusan Islands (*melanurus*, *opisthochrus*, *hypolizus*), and (vii) mainland tropical Asia (*malabaricus*, *tricolor*, *macrourus*), with oriental magpie-robin as an outgroup (Korneliussen et al. 2014). We extracted the best 10 kb loci using a 50 kb sliding window across the eight groups (including outgroup), ensuring all groups are represented at each locus and there is no more than 13% missingness. The taxon *javanus* was removed due to poor sequence quality. In total, we extracted 3,713 sequences for each taxon and aligned them using MAFFT (Katoh et al. 2002).

We created a sequence-based maximum likelihood tree with these loci using the same parameters as the SNP tree.

In addition, we created two subsets containing 1,000 and 5,000 SNPs, respectively, from the SNP dataset to construct a species tree using SNAPPER v1.0.2 (Stoltz et al. 2020). These subsets were chosen in light of the immense computational requirement of these methods. The log files were checked in Tracer to confirm that a sufficient number of MCMC chains had been run (Rambaut et al. 2018). A summary tree was obtained using TreeAnnotator (Drummond and Rambaut 2007).

The maximum likelihood tree suggested the presence of incomplete lineage sorting or introgression in certain taxa (see Results), prompting us to investigate secondary gene flow using qpBrute (Leathlobhair et al. 2018; Liu et al. 2019). We reduced the dataset to certain groups of interest due to computational limitations of qpBrute. These groups are (i) the East Java clade (*omissus, javanus*), (ii) Kangean Island (*nigricauda*), (iii) Sabah (*stricklandii*), (iv) remaining Borneo (*suavis*), and (v) mainland tropical Asia (*malabaricus*, *tricolor*, *macrourus*) with the oriental magpie-robin as an outgroup. These samples were remapped to the Collared Flycatcher *Ficedula albicollis* genome for chromosomal information (RefSeq accession: GCF_000247815.1). We then called SNPs again only for the major chromosomes before removing linked SNPs with a pairwise linkage disequilibrium correlation coefficient higher than 0.1. The resulting 463,621 SNPs were inputted into qpBrute for fitting admixture graphs. Bayes factors for each admixture graph were then calculated with a burnin of 1,800,000 and 9,000,000 iterations. Convergence was determined using the Gelman-Rubin convergence diagnostic and likelihoods calculated. A difference in likelihood values <3 was used as a benchmark for rejecting models.

### Population Genomic Analysis

We recalled 2,579,997 SNPs for 51 shama samples in ANGSD, without the outgroup (-uniqueOnly 1, -remove_bads 1, -only_proper_pairs 1, -SNP_pval 1e-6, -trim 5, -minMapQ 30, -minQ 30, -minMaf 0.05, -minIndDepth 3, -geno_mindepth 3, 90% presence across individuals). We assessed population division in the radiation by running PCA in PCAngsd (Meisner and Albrechtsen 2018) and admixture in NGSadmix up to *K* = 15 (Skotte et al 2013). Population differentiation and diversity statistics (*π*, *F*_ST_, *D*_XY_) were calculated using pixy (Korunes and Samuk 2021).

### Mitogenomic Analysis

We also harvested the mitogenome from our samples by mapping the reads to the complete mitogenome of *C. saularis* (GenBank Accession: NC_030603) using BWA–MEM during initial filtering. We employed ANGSD to call consensus sequences for each sample using -doFasta 3 (Korneliussen et al. 2014). These sequences were screened by ORF Finder (https://www.ncbi.nlm.nih.gov/orffinder/) to ensure the same orientation across all sequences and the absence of nuclear genes that may have been mistakenly included in the extraction. Following a quality check, we managed to retain all taxa including *barbouri* and *albiventris*.

MAFFT was used to align the mitochondrial sequences, and samples with more than 30% missingness were removed (Katoh et al. 2002). We calculated pairwise divergences among the taxa with MEGAX (Kumar et al. 2018). A maximum likelihood tree was constructed using RAxML-NG using the GTR + I + G model with 100 bootstraps (Stamatakis 2014).

### Calculating Evolutionary Distinctness

Given the massive size of the generated sequence dataset (∼297 Mb), Bayesian approaches proved too computationally intensive and time consuming to run. We instead opted to use a non-Bayesian dating method, RelTime-OLS, using a relaxed clock as implemented in MEGAX that is known to be fast and accurate (Mello 2018; Tao et al. 2020). We then calculated ED, PD, and Evolutionarily Distinct and Globally Endangered (EDGE) scores following Jetz et al. (2014). We also calculated the loss in PD using ED/PD*100. To calculate EDGE scores, a conservation status needs to be assigned to each taxon; yet the IUCN Red List does not provide conservation status assessments for taxa that are not considered at the species level according to its internal taxonomy. In the case of the white-rumped shama complex, it has assigned a “Least Concern” status to the two species it recognizes (white-rumped shama *C. malabaricus* and Andaman shama *C. albiventris*). Therefore we assigned conservation status assessments to each of the lineages ourselves, using the IUCN status criteria and the best field knowledge of the day. For example, within the Barusan clade (including taxa *hypolizus, melanurus, opisthochrus* and unnamed populations on surrounding islands), most populations are now considered extinct in the wild, with the exception of *melanurus* on Siberut, which was still found in single digits in 2018 (Rheindt et al. 2019). The East Javan taxon *omissus* is presently known from only one national park in the wild and is under intense danger to become extinct in the wild because of rampant poaching and trade (Chng and Eaton 2016). As such, we decided to group the above-mentioned clades as “Critically Endangered.” The last few expeditions to the Kangean islands have not detected a sustainable population of *nigricauda* in the wild (B. van Balen, personal communication, July 2015). Only a single individual was heard during the last expedition, possibly the last remaining individual on this small island (Irham 2016). Therefore we opt to classify *nigricauda* as “Critically Endangered” for the purpose of our calculations, although it is quite possibly extinct in the wild.

### Comparing Population Dynamics Across Time

In the four taxa that were represented by both historic and modern samples—*macrourus*, *suavis*, *melanurus*, and *hypolizus—*we tracked population-genetic parameters over the course of a century. In each of these taxa and time cohorts, the sample with the best DNA sequence quality was chosen if multiple individuals were available, with male samples being prioritized. The coverage and missingness across each sample are comparable to rule out potential biases.

We conducted the following analyses 1-on-1 per taxon to minimize the effect of differing population sizes on our results. We carried out variant calling using ANGSD (-uniqueOnly 1 -remove_bads 1 -only_proper_pairs 1 -minMapQ 30 -minQ 20 -minInd 2 -setMinDepth 3 -SNP_pval 1e-6 -skipTriallelic 1) for each taxon. We used the script popgenWindows.py (https://github.com/simonhmartin/genomics_general) to calculate nucleotide diversity, *F*_ST_ and *D*_XY_ of the historic population and modern population using a window size of 50 kb and a step size of 10 kb. Furthermore, we calculated heterozygosity values for each sample using ANGSD based on a folded site frequency spectrum estimation.

In order to search for ROH, we remapped the same samples to the Collared Flycatcher *F. albicollis* genome for chromosomal information and better N50 and L50 statistics that may aid in retrieving longer ROHs (RefSeq accession: GCF_000247815.1). We generated genomic vcf files using bcftools and the above-mentioned reference genome. PLINK was used to calculate ROHs for each taxon and time cohort following the recommended filters (–homozyg-density 25 –homozyg-gap 500 –homozyg-het 15 –homozyg-window-snp 50 –homozyg-window-het 15 –homozyg-window-missing 1 –homozyg-window-threshold 0.05 –homozyg-snp 50 –homozyg-kb 300) (Purcell et al. 2007; Meyermans et al. 2020).

## Supplementary Material

msac189_Supplementary_Data

## Data Availability

Raw FASTQ files of whole-genome re-sequenced samples are available on NCBI Sequence Read Archive under BioProject no.: PRJNA867483.
