# Supplementary material for: Genomes From Historic DNA Unveil Massive Hidden Extinction and Terminal Endangerment in a Tropical Asian Songbird Radiation
Source: Mol Biol Evol. 2022 Sep 6;39(9):msac189. doi: 10.1093/molbev/msac189 (PMC9486911; doi:10.1093/molbev/msac189)
Supplement: msac189_Supplementary_Data [file msac189_supplementary_data.zip › Shama_SI.pdf]

Supplementary Materials

Taxonomic revision of the *Copsychus malabaricus* complex

Here we present a taxonomic revision of the white-rumped shama *C. malabaricus* complex (not including the Andaman shama *C. albiventris*) based on current literature and new information generated in this study. Species are classified into three categories: (1) Extinct, possibly extinct or extinct-in-the-wild; (2) Terminally endangered; and (3) Other species of concern. Our taxonomic revision is guided by the widely-applied multi-dimensional Biological Species Concept (Mayr 1968, 1992).

1) Extinct, possibly extinct or extinct-in-the-wild species

Kangean shama *C. nigricauda* (monotypic): Probably extinct in the wild, the Kangean shama has always constituted a taxonomic puzzle because of its unusual melanistic tail coloration (Vorderman 1893), setting it apart from all surrounding populations. The stability of this trait is surprising given that the Kangean islands would have been connected to Java and Borneo as recently as ~10,000 years ago (Voris 2000), facilitating contact with the different-looking adjacent populations of *omissus* (East Java) and *suavis* (southern Kalimantan), both of which show much white in the tail and present additional plumage differences. Given the likely reproductive importance of the white tail flash in inter-individual signalling, the maintenance of this trait despite recent land connections to dissimilar populations on two geographic fronts corroborates the surprisingly distinct placement in the genomic tree (fig. 1). This phylogenetic position is coupled with levels of mtDNA divergence that are consistent with species level (fig. 1 and supplementary tables S3-5). A handful of individuals recently found in Javan markets (exact details withheld) exhibit morphological characters consistent with this taxon (fig. 1, third photo from the left), and their taxonomic identity will hopefully soon be tested against the historic genome reported in this study, affording a glimmer of hope for the captive survival of the species.

Maratua shama *C. barbouri* (monotypic): Long kept at subspecies level, the Maratua shama has recently been proposed for species status on the basis of its fairly distinct mtDNA divergence and distinct combination of crown and tail coloration (Chua et al. 2015), which arguably render it one of the morphologically most outstanding taxa in the complex. This treatment was followed by some modern authorities (Eaton et al. 2016, 2021). We did not have access to its genomic DNA in this study, but support its species status pending the

acquisition and analysis of good genomic DNA. The Maratua shama has not been encountered in recent comprehensive surveys of this small island (Burner et al. 2018), and is presently considered extinct-in-the-wild. A handful of individuals have survived in captivity in a Javan conservation-breeding facility (identity withheld) at the time of writing.

2) Terminally endangered species

Larwo Shama *C. omissus* (including subspecies *javanus*): The two eastern Javan taxa, which have hitherto never been considered an independent species (supplementary table S1), emerged as the genomically most distinct clade in the entire complex (fig. 1). Their distinctness was underscored by mitogenomic data indicating some of the deepest mtDNA divergences found in the entire complex, reaching levels consistent with traditional DNA barcoding thresholds for species status (Hebert et al. 2004; Kerr et al. 2007) (supplementary tables S3-5). Importantly, the mitogenomic DNA suggested a basal position even with respect to the Andaman shama *C. albiventris*, which has long been considered distinct at the species level (supplementary fig. S7). We regard this evidence as supporting species status of these two taxa (*omissus* and *javanus*) as the 'Larwo shama' *C. omissus*. The fact that Larwo shamas have never been considered at the species level by previous classifications is perhaps surprising in hindsight. They have a distinct plumage with a whitish rather than rufous belly (fig. 1) (Eaton et al. 2021). Among Javan bird-keepers, they command the highest prices because of their unusual reproductive behavioural features, which involve the raising of their much shorter crown feathers into a roundish crest during courtship (fig. 1) (Eaton et al. 2021), setting them apart from all other shamas. The name "Larwo" is the local moniker used by Javan bird breeders to refer to this distinct taxon (Bahasa Indonesia: murai batu larwo = "Larwo shama").

3) Other species of concern

White-crowned shama *C. stricklandii* (monotypic): This taxon has undergone a checkered classification history either as a species or subspecies of the white-rumped shama (supplementary table S1). Morphologically, its white rather than black crown instantly sets it apart from all other taxa except Maratua shama *C. barbouri* (although the latter shows pronounced differences in tail coloration from taxon *stricklandii*). Previous genomic inquiries about gene flow dynamics across the Bornean hybrid zone between *stricklandii* and *suavis* have supported species status of the white-crowned shama (Lim et al. 2017). Our genome-tree analysis corroborates this conclusion, showing that *stricklandii* – together with Kangean

shama *C. nigricauda* – holds the most distinct phylogenetic position within the entire *C. malabaricus* complex when Larwo shama *C. omissus* is excluded (fig. 1). A propensity for hybridisation with adjacent *suavis* on Borneo has been documented and studied (Lim et al. 2017) and is here corroborated by our secondary gene flow analyses (fig. 1) showing considerable past genomic contributions from *stricklandii* into *suavis*. However, in the present interglacial which we live in, such gene flow seems to occur at a low level (Lim et al. 2017), and the hybrid zone is sufficiently narrow and stable to allow a diagnosis of the two taxa as belonging to two different species under the Biological Species Complex (Mayr 1968, 1992). While still widespread at the moment, the white-crowned shama is rapidly being trapped out of wide parts of its range and will deserve conservation attention soon.

<u>Sri Lankan Shama *C. leggei* (monotypic)</u>: This Sri Lankan endemic taxon has not been considered distinct at the species level in modern times. It differs from continental Asian white-rumped shama populations – including nominotypical ones from the adjacent Western Ghats region in southern India – in its overall shorter tail, reduced sexual dimorphism and paler belly coloration. These phenotypic distinctions have often been interpreted as mere variations in the white-rumped shama's checkerboard theme of morphological differences, but without paying heed to the realization that Sri Lanka has been continuously connected to southern India during Quaternary cooling cycles, including most recently at only ~10,000 years ago (Voris 2001). The repeated land connections would have afforded opportunities for gene flow between *leggei* and *malabaricus* that is inconsistent with the levels of genomic and mitochondrial diversification evident today (fig. 1, supplementary table S3-5), suggesting that the considerable phenotypic differences of *leggei* would have constituted a reproductive barrier to gene flow during the most recent land bridges. Based on our nuclear genomic and mitogenomic data in combination with the existing phenotypic evidence, we advocate that the Sri Lankan shama's taxonomic status is best reflected at the species level.

<u>White-rumped shama *C. malabaricus* (polytypic)</u>: We recommend for all other taxa to continue treatment as subspecies under the white-rumped shama *C. malabaricus* umbrella species pending further information. This includes *malabaricus* (peninsular India), *macrourus* (monsoon regions of continental Asia), *tricolor* (peninsular Malaysia to West Java), *suavis* (Borneo minus Sabah), *hypolizus* (Simeulue – extinct in the wild; Rheindt et al. 2019), *opisthochrus* (Babi – extinct in the wild and probably also in captivity; Rheindt et al. 2019), *melanurus* (Nias, Siberut), *mirabilis* (Panaitan – extinct), and *ngae* (Langkawi and

western Thai archipelago – possibly extinct in the wild; Wu and Rheindt 2022). However, this arrangement may need to be further adjusted in the future. We see the potential for three future species-level splits within this complex, discussed in sequence:

(1) The distinct taxon *ngae* – recently described to science from Langkawi and other islands on the west coast of the Thai-Malay Peninsula (Wu and Rheindt 2022) – only yielded poor DNA that did not permit inclusion in genomic analysis but allowed mitogenomic comparisons. In its mitochondrial DNA, it emerged as highly distinct (supplementary fig. S7), with a surprising sister position to Andaman Shama *C. albiventris* rather than to classical members of the white-rumped shama complex. Our currently recommended treatment of *ngae* as a subspecies of the white-rumped shama *C. malabaricus* is conservative and follows the rationale advanced in its original scientific description (Wu and Rheindt 2022), but future data may well lead to its elevation to species level (as 'Langkawi shama').

(2) For the taxa which yielded sufficiently good genome-wide DNA for analysis, *suavis* from Borneo emerged as most basal among the taxa here included within the narrowly-circumscribed white-rumped shama *C. malabaricus* (fig. 1), flagging it for future inquiry with regards to potential species status. Morphologically, it greatly resembles the mainland cluster of white-rumped shamas (Eaton et al. 2021), and our demonstrations of secondary gene flow between it and *stricklandii* (see above) suggest that its phylogenetic position on the SNP and sequence-based trees may be impacted by genetic introgression (fig. 1) pulling it out from the continental cluster. Pending further inquiry, we consider it safest to maintain this taxon as a subspecies of white-rumped shama *C. malabaricus*.

(3) The four Barusan taxa *hypolizus, opisthochrus*, *melanurus* and *mirabilis* have recently been considered members of one independent species, the Barusan shama *C. melanurus*, based on conspicuous plumage distinctions (Eaton et al. 2021), but their genomic differentiation from the continental cluster of white-rumped shamas was surprisingly shallow in our analyses, calling for caution regarding their taxonomic rank (fig. 1 and supplementary table S3-5). While we consider their future (re-) elevation to species level possible, we would like to err on the side of caution at the moment and maintain them as a group of subspecies within the white-rumped shama *C. malabaricus*. This conservative taxonomic practice may have negative conservation repercussions, as constituent member taxa of this

complex are either entirely extinct (*mirabilis, opisthochrus*), or extinct-in-the-wild (*hypolizus*), or critically endangered (*melanurus*) (Rheindt et al. 2019).

Table S1. Taxonomic treatment of the *Copsychus malabaricus* complex in various global bird taxonomies. Sp: Taxon recognised at species level, Subsp: Taxon recognised at subspecies level. Taxa recognised at the species level are shown in vivid colours (strong blue: C. *malabaricus*; barred green: C. *barbouri*; red: *C. omissus*; pink: *C. nigricauda*; orange: *C. stricklandii*; purple: *C. albiventris*; bright yellow: *C. leggei*), those at the subspecies level in corresponding fainter hues. Taxa not recognised by particular sources are shown in grey.

Taxon	Peters's (1964) Checklist	HBW/ BirdLife (2019)	Clements's (2019) Checklist	Gill & Rasmussen (2021)	this study
malabaricus	Sp	Sp	Sp	Sp	Sp
interpositus	Subsp				
macrourus		Subsp	Subsp	Subsp	Subsp
tricolor	Subsp	Subsp	Subsp	Subsp	Subsp
omissus	Subsp	Subsp	Subsp		Sp
javanus	Subsp	Subsp	Subsp		Subsp
suavis	Subsp	Subsp	Subsp	Subsp	Subsp
leggei	Subsp	Subsp	Subsp	Subsp	Sp
nigricauda	Subsp	Subsp	Subsp	Subsp	Sp
melanurus	Subsp	Subsp	Subsp	Subsp	Subsp
hypolizus	Subsp	Subsp	Subsp		Subsp
opisthochrus	Subsp	Subsp	Subsp		Subsp
mirabilis		Subsp	Subsp	Subsp	Not sampled
ngae					Subsp
stricklandii	Sp	Subsp	Subsp	Sp	Sp
barbouri	Subsp	Subsp	Subsp	Subsp	Sp
albiventris	Subsp	Sp	Sp	Sp	Sp

Table S2. List of samples sequenced in this study. Abbreviations: LKCNHM: Lee Kong Chian Natural History Museum (Singapore), AMNH: American Museum of Natural History (New York), RMNH: Naturalis Biodiversity Center (Leiden), SMNH: Swedish Natural History Museum (Stockholm), MNHN: Muséum national d'Histoire naturelle (Paris), AEL: samples collected for this study (ultimately deposited at the LKCNHM). "Skin" refers to toepad samples from museum skins, "fresh" refers to blood or breast muscle tissue samples kept in ethanol or other buffers under refrigeration.

**Specimen ID/Voucher number**	**Taxon name and population identity of *Copsychus* sample**	**Specimen type**	**Collection year**	**Holding institution**	**Collecting locality**
ZRC 3.22575	Sumatra *tricolor*	skin	1927	LKCNHM	Telok Pandji, Kota Pinang, Sumatra, Indonesia
ZRC 3.22599	Maratua *barbouri*	skin	-	LKCNHM	Maratua Island, East Kalimantan, Indonesia
ZRC 3.22474	Sipora *melanurus*	skin	1924	LKCNHM	Sipora Island, West Sumatra, Indonesia
ZRC 3.22482	East Java *omissus*	skin	1920	LKCNHM	Badjoelmati, East Java at 100ft, Indonesia
ZRC 3.4175	Central Java *javanus*	skin	1920	LKCNHM	Karangbolang, Central Java at 100ft, Indonesia
ZRC 3.22478	Anambas *tricolor*	skin	1925	LKCNHM	Pulau Siantan, Anambas Islands, Indonesia
ZRC 3.22515	Peninsular Malaysia *ngae*	skin	1916	LKCNHM	Pulau Dayang Bunting, Langkawi, Malaysia
ZRC 3.22511	Peninsular Malaysia *ngae*	skin	1916	LKCNHM	Telok Waw/ Wau, Terutau Island, Thailand

ZRC 3.22594	Natuna *tricolor*	skin	1950	LKCNHM	Pulau Redang, Natuna Islands, Indonesia
SKIN 580290	Andaman *albiventris*	skin	1906	AMNH	Mt. Harriet, Port Blair, South Andaman Islands, India
SKIN 580291	Andaman *albiventris*	skin	1906	AMNH	Banatong I., Middle Andaman Islands, India
SKIN 580388	Kangean *nigricauda*	skin	-	AMNH	Kangean Island, Indonesia
SKIN 462930	West India *malabaricus*	skin	1938	AMNH	Jagalbed, Bombay, India
SKIN 291682	Vietnam *macrourus*	skin	1930	AMNH	Hoi Xuan, Vietnam
SKIN 447772	Kalimantan *suavis*	skin	1935	AMNH	Parit, (Tjempaga), Sampit, S Borneo, Indonesia
SKIN 447773	Kalimantan *suavis*	skin	1935	AMNH	Riam (Kotawaringin), SW Borneo, Indonesia
SKIN 580368	East Java *omissus*	skin	1886	AMNH	Lawang, Java, Indonesia
SKIN 344447	West India *malabaricus*	skin	1939	AMNH	Antarsante, S.W. Mysore State, India
RMNH.AVES.129477	Sri Lanka *leggei*	skin	-	RMNH	Pintenne, Sri Lanka
RMNH.AVES.129560	probably Simeulue, *hypolizus*	skin	1913	RMNH	Ajer Dingin, Indonesia; Inferred to be from Simeulue
RMNH.AVES.129549	probably Nias, *melanurus*	skin	1895	RMNH	H. Madjeio, Indonesia; Inferred to be from Nias

RMNH.AVES.129554	probably Nias, *melanurus*	skin	1896	RMNH	Lahago, Indonesia; Inferred to be from Nias
SEA015	Sarawak *suavis*	fresh	1997	Burke Museum	Kubah National Park, Kuching, Sarawak, Malaysia
NRM 20036774	Vietnam *macrourus*	fresh	-	SMNH	Vietnam
NRM 20036776	Vietnam *macrourus*	fresh	-	SMNH	Vietnam
MNHN 33-6D (JF163)	Laos *macrourus*	fresh	-	MNHN	Ban Hathin, Phongsaly, Laos
MNHN 33-05F (JF200)	Laos *macrourus*	fresh	-	MNHN	Ban Sopkhang, Phongsaly, Laos
MNHN JF318	Cambodia *macrourus*	fresh	-	MNHN	Site 1 after Andom Vei, Siem Reap, Cambodia
MNHN JF401 (Voucher no. CG 2013-194)	Laos *macrourus*	fresh	-	MNHN	Ban Donkham, Luang Prabang, Laos
MNHN JF409 (Voucher no. CG 2013-196)	Laos *macrourus*	fresh	-	MNHN	Ban Long Khan, Xiengkhouang, Laos
MNHN JF411	Laos *macrourus*	fresh	-	MNHN	Ban Long Khan, Xiengkhouang, Laos
MNHN JF1793	Vietnam *macrourus*	fresh	-	MNHN	Hon Chong, hill of Binh Trih, Kieng Giang, Vietnam

MNHN JF1795	Vietnam *macrourus*	fresh	-	MNHN	Hon Chong, hill of Binh Trih, Kieng Giang, Vietnam
MNHN JF4245	Vietnam *macrourus*	fresh	-	MNHN	Cuc Phuong National Park, Ninh Binh, Vietnam
MNHN JF4287	Vietnam *macrourus*	fresh	-	MNHN	Me Linh Biodiversity Center, IEBR, Vinh Phuc, Vietnam
MNHN 05-37	Laos *macrourus*	fresh	-	MNHN	Nam Kan-Nam Ngao, Houay Xay, Bokeo, Laos
MNHN 04-8F	Thailand *macrourus*	fresh	-	MNHN	Korat, Nakhon Ratchasima, Thailand
MNHN 06-106 (Voucher no. MNHN CG 2000-0337)	Thailand *macrourus*	fresh	-	MNHN	Kapoe, Ranong, Thailand
KK03	Burma *macrourus*	fresh	2017	AEL	Salween river, Burma
KK11	Burma *macrourus*	fresh	2017	AEL	Salween river, Burma
DOT 15021	Sabah *stricklandii*	fresh	2005	AMNH	Mendolong, ca. 31 km SE Sipitang, Sabah, Malaysia
DOT 334	Sabah *stricklandii*	fresh		AMNH	Kinabalu Park, Sabah, Malaysia
SIM39	Simeulue *hypolizus*	fresh	2018	AEL	Simeulue Island, Indonesia
SIM43	Babi *opisthochrus*	fresh	2019	AEL	Babi Island, Indonesia
SIM44	Simeulue *hypolizus*	fresh	2019	AEL	Simeulue Island, Indonesia
SIM45	Nias *melanurus*	fresh	2019	AEL	Nias Island, Indonesia

SIM46	probably Nias, *melanurus*	fresh	2019	AEL	Aceh (market), Indonesia
SIM47	undescribed population, Banyak Island	fresh	2019	AEL	Banyak Island, Indonesia
NIA01	Nias *melanurus*	fresh	2019	AEL	Onolimbu, Nias Island, Indonesia
NIA02	Nias *melanurus*	fresh	2019	AEL	Onolimbu, Nias Island, Indonesia
NIA75	Nias *melanurus*	fresh	2019	AEL	Gunung Sitoli, Nias Island, Indonesia
SIB35	Siberut *melanurus*	fresh	2019	AEL	Matotonan, Siberut Island, Indonesia
PBI-MAL-052	Peninsular Malaysia *tricolor*	fresh	2010	AEL	Ulu Gombak, Selangor, Malaysia
PBI-MAL-033	Peninsular Malaysia *tricolor*	fresh	2010	AEL	Ulu Gombak, Selangor, Malaysia
L1739	Singapore *tricolor*	fresh	2015	AEL	Singapore
L2328	Singapore *tricolor*	fresh	2015	AEL	Singapore
L1547	Singapore *tricolor*	fresh	2013	AEL	Singapore
L2229	Singapore *tricolor*	fresh	2018	AEL	Singapore
L2241	Singapore *tricolor*	fresh	2019	AEL	Singapore
JBPC2740	Singapore *tricolor*	fresh	-	AEL	Singapore

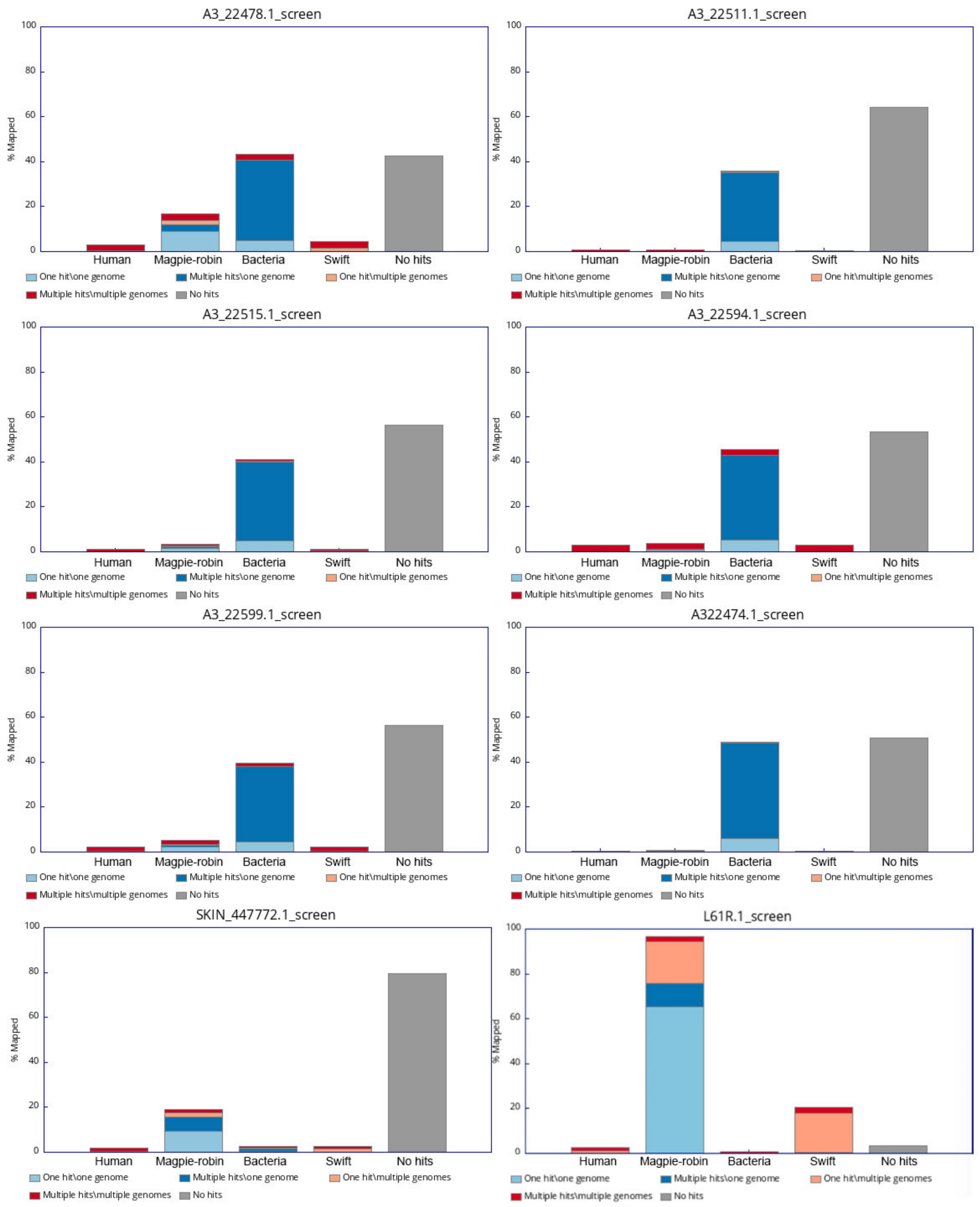

Fig. S1. Visualisation of Fastq_screen results for read 1, showing the seven samples removed due to high 'no hits' mapping plus one successful sample (L61R; bottom right) which contained a high volume of reads mapping to the Seychelles magpie robin *Copsychus sechellarum* (abbreviated as 'Magpie-robin') that were eventually filtered out and used for downstream analyses.

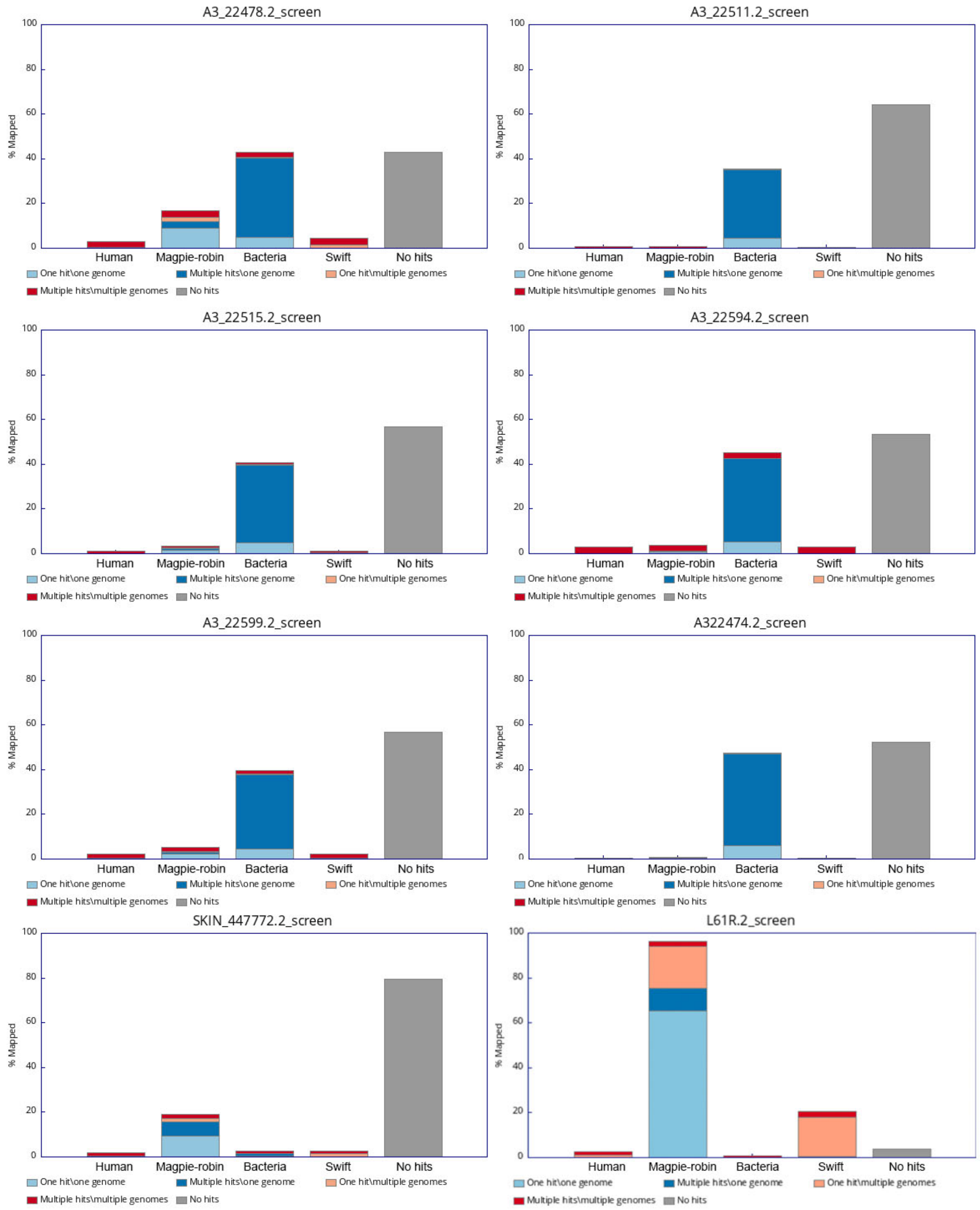

Fig. S2. Visualisation of Fastq_screen results for read 2, showing the seven samples removed due to high 'no hits' mapping plus one successful sample (L61R; bottom right) which contained a high volume of reads mapping to the Seychelles magpie robin *Copsychus sechellarum* (abbreviated as 'Magpie-robin') that were eventually filtered out and used for downstream analyses.

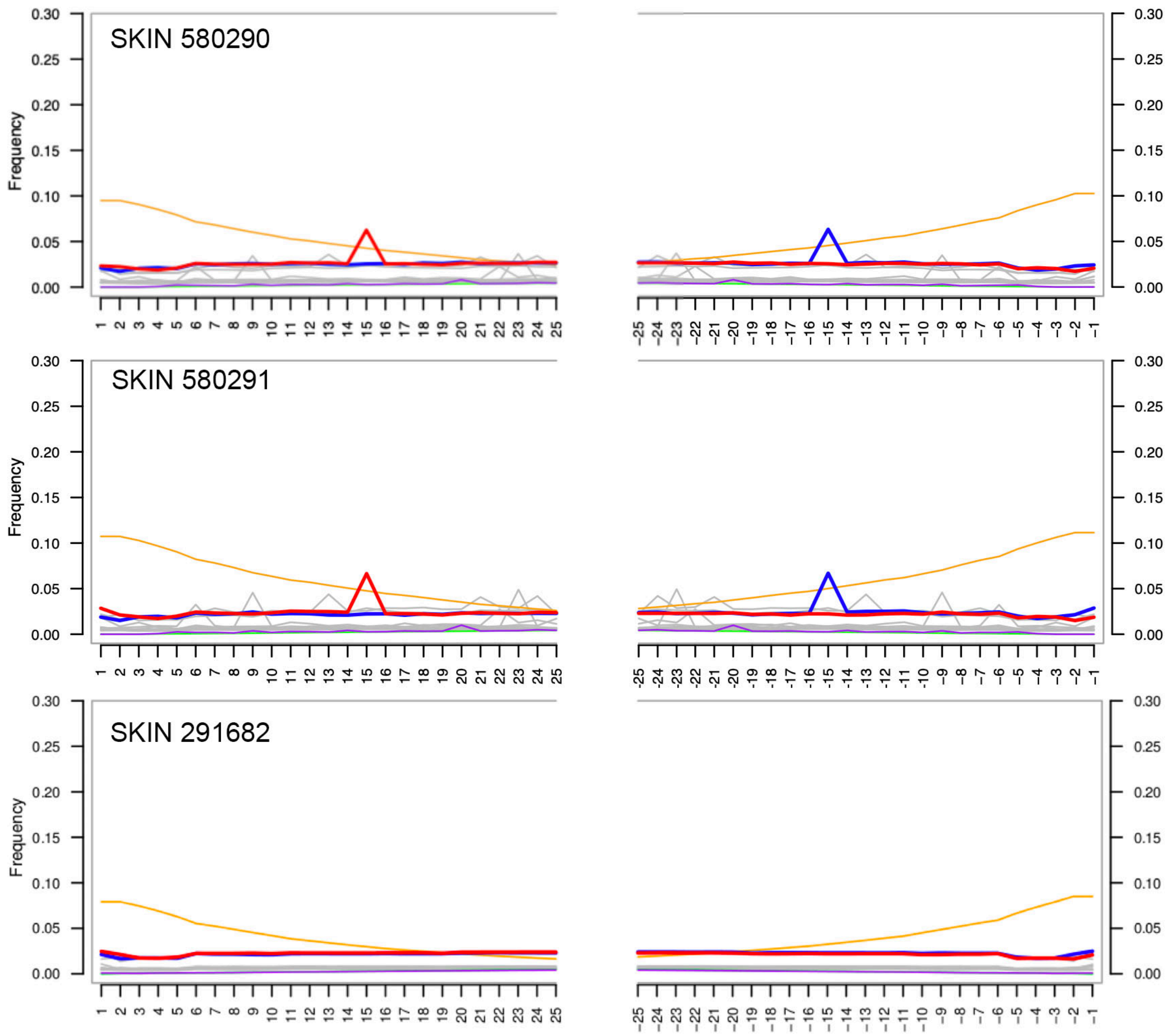

Fig. S3. Summary report from mapDamage visualising *post mortem* DNA damage for two Andaman shama samples (top two) and one *macrourus* sample (bottom). High levels of C to T substitutions and G to A substitutions in the Andaman shamas compared to the *macrourus* sample justified their removal from the dataset. Red: C to T substitutions, Blue: G to A substitutions, Grey: All other substitutions, Orange: Soft-clipped bases.

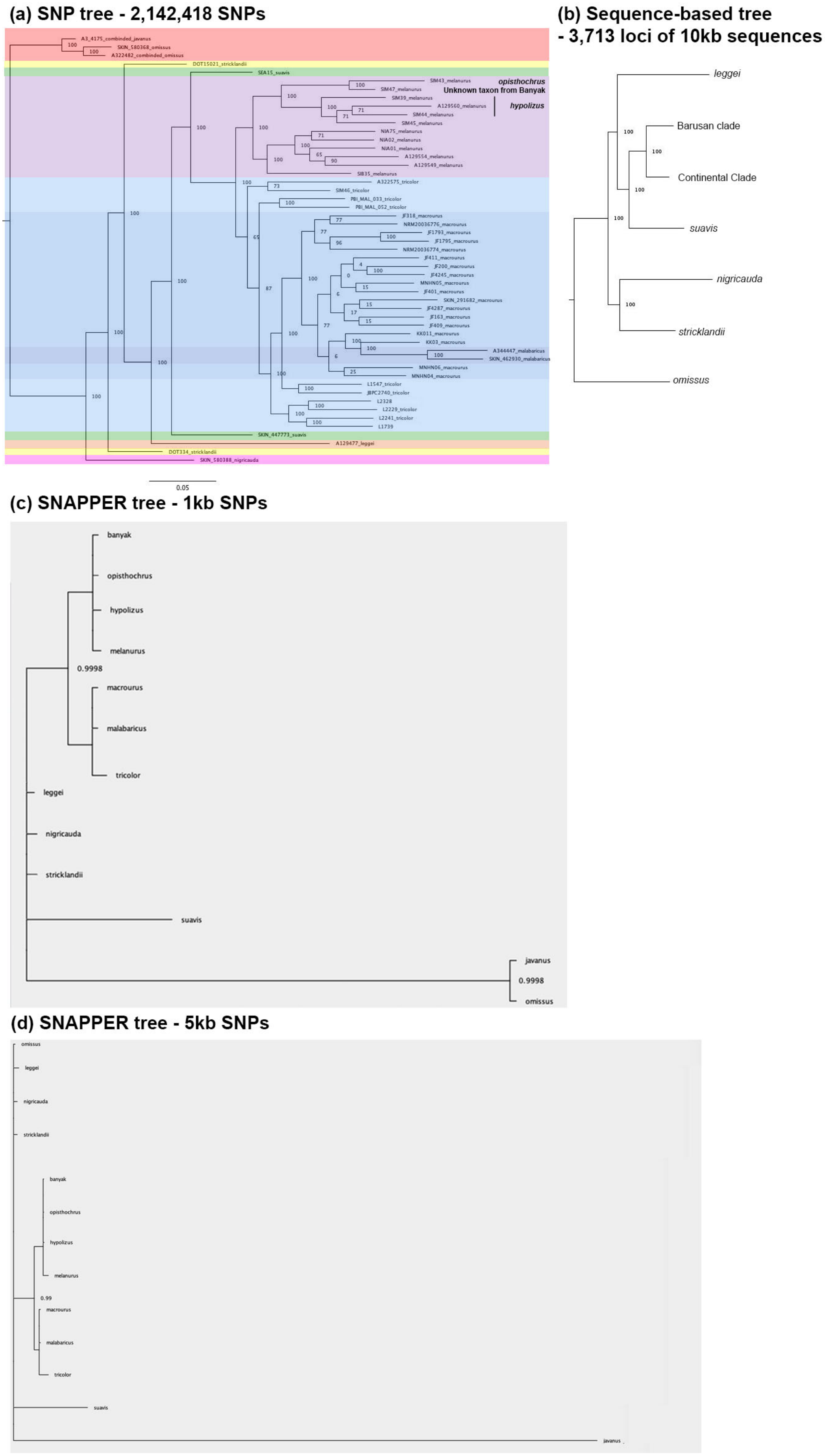
(a) SNP tree - 2,142,418 SNPs
opisthochrus
Unknown taxon from Banyak
hypolizus
0.05
(b) Sequence-based tree
- 3,713 loci of 10kb sequences
leggei
Barusan clade
Continental Clade
suavis
nigricauda
stricklandii
omissus
(c) SNAPPER tree - 1kb SNPs
banyak
opisthochrus
hypolizus
melanurus
0.9998
macrourus
malabaricus
tricolor
leggei
nigricauda
stricklandii
suavis
javanus
omissus
(d) SNAPPER tree - 5kb SNPs
omissus
leggei
nigricauda
stricklandii
banyak
opisthochrus
hypolizus
melanurus
0.99
macrourus
malabaricus
tricolor
suavis
javanus

Fig. S4. (a) Maximum likelihood SNP tree obtained from 51 whole genome resequenced samples (2,142,418 SNPs) using maximum-likelihood RAxML with 100 bootstrap replicates. Bootstrap values provided at each node. Colours refer to taxon information in fig. 1. (b) Maximum likelihood sequence-based tree obtained from consensus fasta for each monophyletic clade among 51 whole genome resequenced samples (3,713 sequence loci spanning 10kb each) using maximum-likelihood RAxML with 100 bootstrap replicates. Bootstrap values provided at each node. (c) – (d) Species trees obtained from 51 whole genome resequenced samples (1,000 SNPs and 5,000 SNPs, respectively) using SNAPPER. Branch support <99% posterior probability was collapsed. Oriental magpie robin *Copsychus saularis* is used as an outgroup in all phylogenetic tree reconstruction.

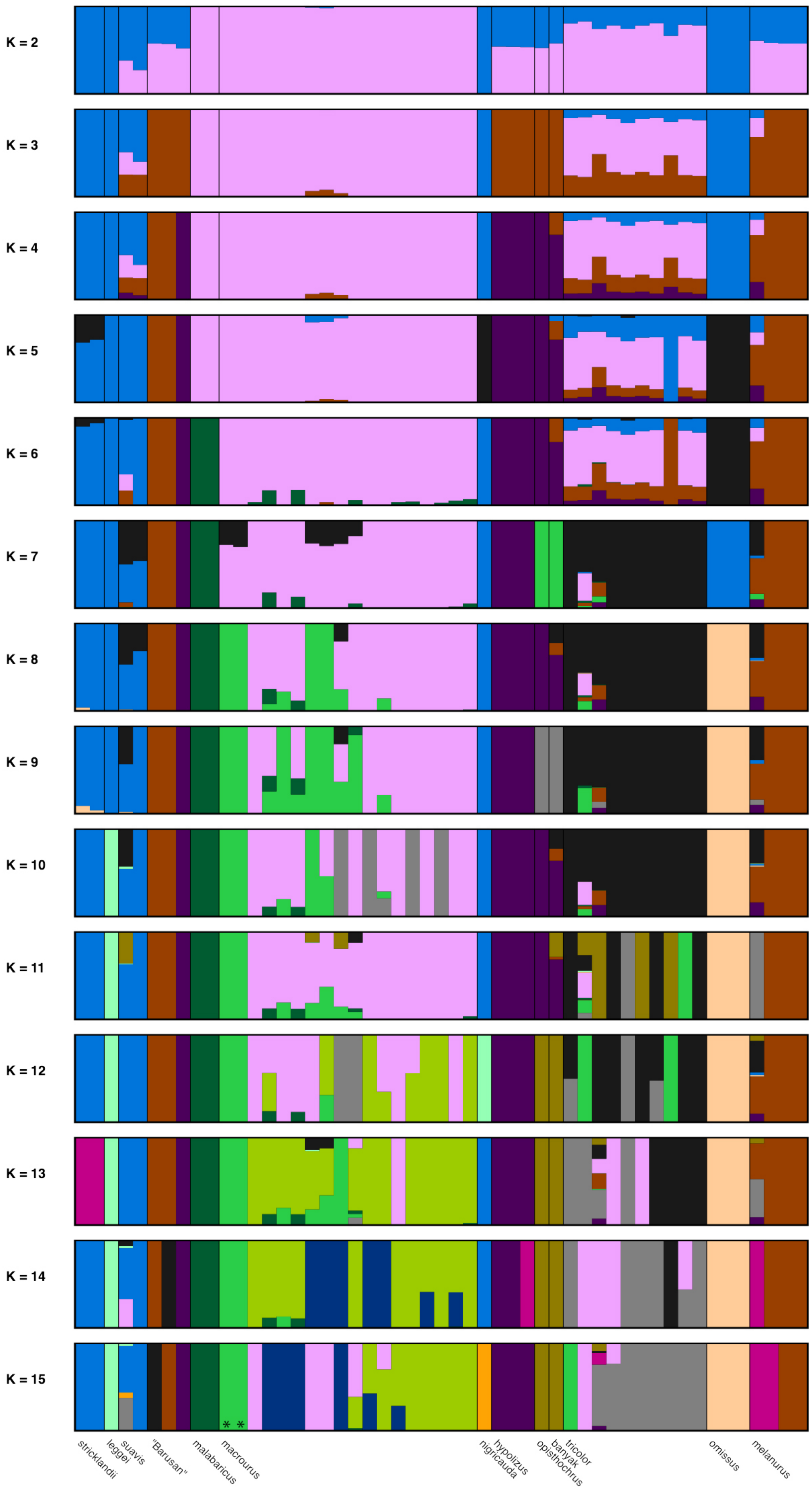

K = 2
K = 3
K = 4
K = 5
K = 6
K = 7
K = 8
K = 9
K = 10
K = 11
K = 12
K = 13
K = 14
K = 15
* *
stricklandii
leggei
suavis
"Barusan"
malabaricus
macrourus
nigricauda
hypolizus
opisthochrus
banyak
tricolor
omissus
melanurus

Fig. S5. ADMIXTURE results of 51 shama samples based on 2,579,997 SNPs using NGSadmix from $K = 2$ to $K = 15$. The bars labelled as "Barusan" refer to museum samples collected from the West Sumatran islands for which exact locality information was not available. Asterisks indicate two *macrourus* individuals from Vietnam further discussed in the text. The best K was found to be $K = 3$.

Table S3. Pairwise mitochondrial divergences calculated from the mitogenomic alignment (15,660 bp) using raw *p* distance.

Please see attached Excel titled Table S3.

Table S4. Pairwise mitochondrial divergences calculated from the COI alignment (707 bp) using raw *p* distance.

Please see attached Excel titled Table S4.

Table S5. Pairwise mitochondrial divergences calculated from the cyt-*b* alignment (1,197 bp) using raw *p* distance.

Please see attached Excel titled Table S5.

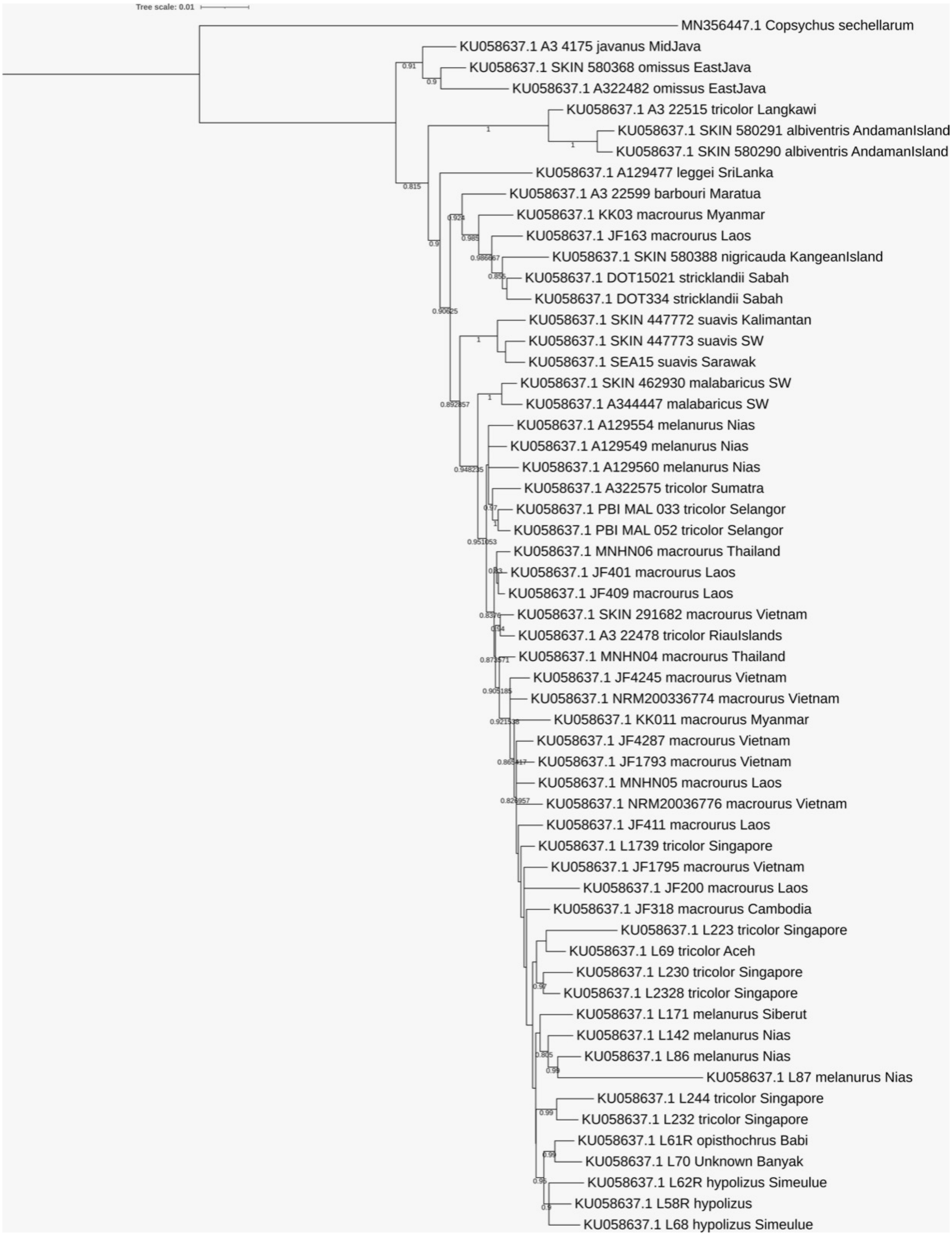

Fig. S6. Maximum Likelihood phylogenetic tree inferred from the mitogenomic dataset (15,660 bp), rooted with the *Copsychus sechellarum* mitogenome as an outgroup. Only bootstrap values above 0.8 are displayed on respective nodes. The sole sample of the newly described taxon *ngae* from Langkawi Island (peninsular Malaysia; Wu and Rheindt 2022) is

labeled as "*tricolor* Langkawi" and emerged as sister to the two samples of *C. albiventris* from the Andaman Islands.

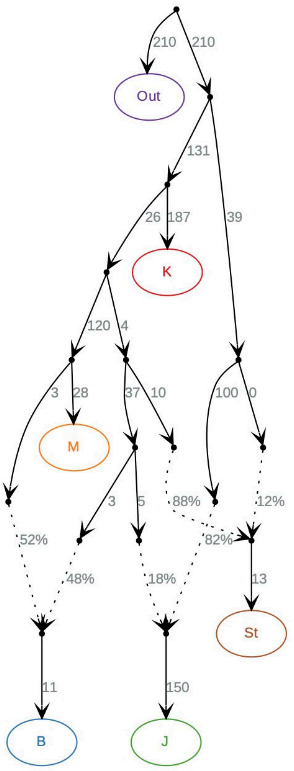
210
210
Out
131
39
26
187
K
120
4
100
0
3
28
37
10
M
3
5
88%
12%
52%
82%
48%
18%
13
St
11
150
B
J

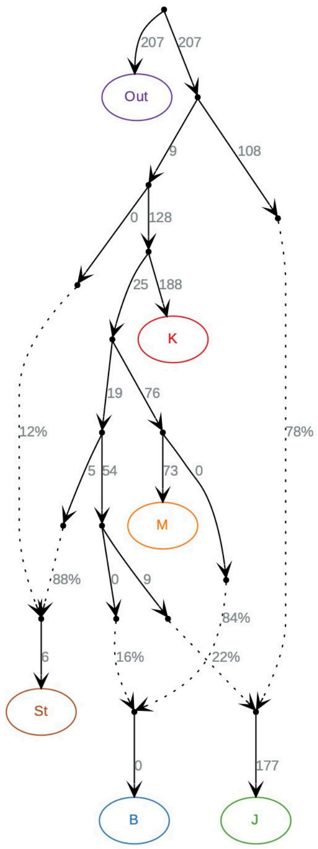
207
207
Out
9
108
0
128
25
188
K
12%
19
76
78%
5
54
73
0
M
88%
0
9
84%
6
16%
22%
St
0
177
B
J

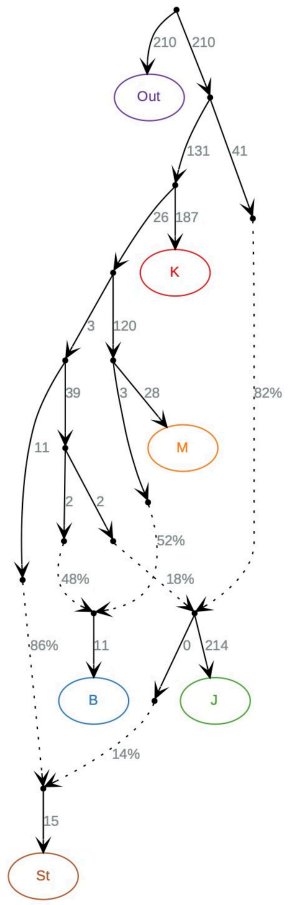
210
210
Out
131
41
26
187
K
3
120
39
3
28
82%
11
M
2
2
52%
48%
18%
11
0
214
86%
B
J
14%
15
St

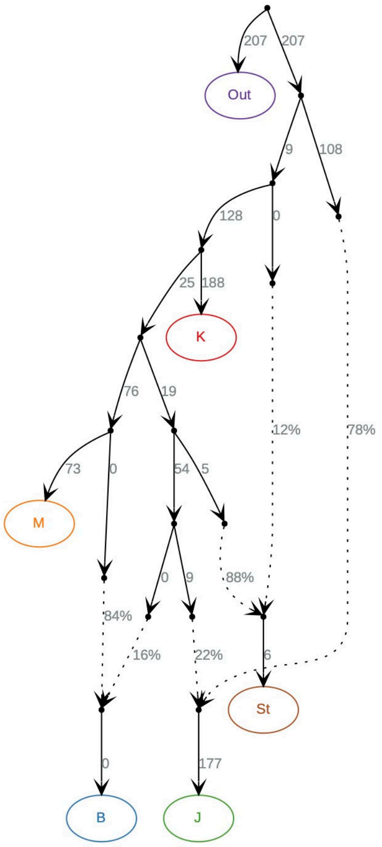
207
207
Out
9
108
128
0
25
188
K
76
19
12%
78%
73
0
54
5
M
0
9
88%
84%
16%
22%
6
St
0
177
B
J

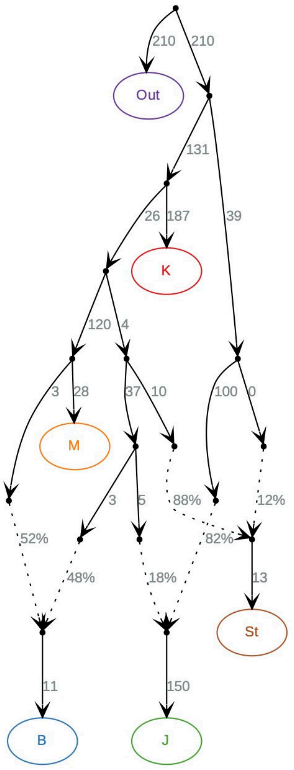
210
210
Out
131
26
187
39
K
120
4
3
28
37
10
100
0
M
88%
12%
3
5
52%
82%
48%
18%
13
St
11
150
B
J

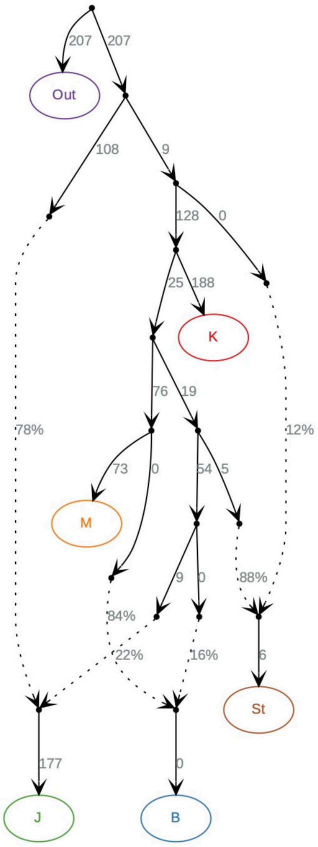
207
207
Out
108
9
128
0
25
188
K
76
19
78%
12%
73
0
54
5
M
9
0
88%
84%
22%
16%
6
St
177
0
J
B

Fig. S7. Admixture graphs based on qpBrute analysis with 463,621 SNPs. Abbreviations: Out: Oriental magpie-robin (*Copsychus saularis*), J: East Java clade (*omissus, javanus*), M: Continental tropical Asian clade (*malabaricus, tricolor, macrourus*), B: Borneo excluding Sabah (*suavis*), St: Sabah (*stricklandii*), K: Kangean Island (*nigricauda*).

Table S6. Heterozygosity values of threatened passerine birds taken from literature. Conservation status abbreviations: CR – Critically Endangered; EN – Endangered; VU – Vulnerable; NT – Near Threatened.

Species	Conservation status	Heterozygosity	Source
Chatham robin<br>*Petroica traversi*	EN	0.39 based on 8 loci	Forsdick et al. 2017
Rarotonga monarch<br>*Pomarea dimidiata*	VU	0.65 based on 7 loci	Chan et al. 2011
Bali starling<br>*Leucopsar rothschildi*	CR	0.47 based on 8 loci	Ogata et al. 2020
Seychelles magpie-robin<br>*Copsychus sechellarum*	EN	0.00015 (genome-wide value)	Cavill et al. 2021

Table S7. Genome-wide heterozygosity values of birds taken from literature. Conservation status abbreviations: CR – Critically Endangered; EN – Endangered; VU – Vulnerable; NT – Near Threatened; LC – Least Concern. The Kakapo value reflects a re-wilded population under intense management, while all other values reflect wild populations to the best of our knowledge.

Species	Conservation status	Heterozygosity	Source
Kakapo<br>*Strigops habroptilus*	CR	~0.05 – 0.09	Dussex et al. 2021
Kea<br>*Nestor notabilis*	EN	0.00091	Li et al. 2014
Crested ibis<br>*Nipponia nippon*	EN	0.00043	Li et al. 2014
Rowi kiwi	VU	~0.001	Prasad et al. 2021

Apteryx rowi			
Dalmatian pelican *Pelecanus crispus*	NT	0.0006	Li et al. 2014
Little egret *Egretta garzetta*	LC	0.00251	Li et al. 2014
Great cormorant *Phalacrocorax carbo*	LC	0.00139	Li et al. 2014
Budgerigar *Melopsittacus undulatus*	LC	0.00431	Li et al. 2014
Bald eagle *Haliaeetus leucocephalus*	LC	0.00043	Li et al. 2014
White-tailed eagle *Haliaeetus albicilla*	LC	0.0004	Li et al. 2014
Turkey vulture *Cathartes aura*	LC	0.00118	Li et al. 2014

Table S8. Summary information for all runs of homozygosity (ROH) harvested. PHOM: proportion of homozygous sites, PHET: proportion of heterozygous sites.

	**Chromosome**	**Starting Position**	**Ending Position**	**Length of ROH (KB)**	**No. of SNPs**	**Average SNP density**	**PHOM**	**PHET**
macrourus Present (NRM-20036776)	1	567278	930580	363.303	11958	0.03	0.999	0.001
	1A	285646	602695	317.05	9827	0.032	0.998	0.002
	1A	74630496	74937899	307.404	5799	0.053	0.998	0.002
	5	113692	434000	320.309	12954	0.025	0.999	0.001
	8	31562478	31865335	302.858	13798	0.022	0.999	0.001
	21	6028824	6386106	357.283	8518	0.042	0.998	0.002
	21	6386107	6832427	446.321	14788	0.03	0.999	0.001
	22	3802546	4206075	403.53	21913	0.018	0.999	0.001
	25	424288	922106	497.819	10440	0.048	0.999	0.001
	25	922107	1413674	491.568	9528	0.052	0.998	0.002
	27	4334070	4703526	369.457	8996	0.041	0.998	0.002
	28	3948810	4358455	409.646	9094	0.045	0.998	0.002
	Z	53416841	53858964	442.124	16029	0.028	0.999	0.001

	Z	55440857	55771195	330.339	19853	0.017	0.999	0.001
	unlocalised scaffold	3113	633438	630.326	17104	0.037	0.999	0.001
	Linkage Group LG34	32382	462309	429.928	7161	0.06	0.998	0.002
	Linkage Group LGE22	43668	582595	538.928	14184	0.038	0.999	0.001
macrourus Past (SKIN 291682)	unlocalised scaffold	323893	1178745	854.853	2571	0.332	0.994	0.006
suavis Present (SEA15)	1	647219	1052980	405.762	17757	0.023	0.999	0.001
	1A	576153	902883	326.731	9296	0.035	0.998	0.002
	1A	74451987	74887170	435.184	7811	0.056	0.998	0.002
	4	28086069	28410543	324.475	18306	0.018	0.999	0.001
	4A	448880	783699	334.82	13307	0.025	0.999	0.001
	5	251120	583186	332.067	17191	0.019	0.999	0.001
	21	6008848	6515595	506.748	12707	0.04	0.999	0.001
	25	424516	987391	562.876	10262	0.055	0.999	0.001
	25	987392	1334436	347.045	3326	0.104	0.995	0.005
	27	4126405	4704787	578.383	15171	0.038	0.999	0.001
	28	3900954	4356149	455.196	10084	0.045	0.999	0.001
	Z	53416072	53849572	433.501	15413	0.028	0.999	0.001
	Z	55133462	55453612	320.151	13500	0.024	0.999	0.001
	unlocalised scaffold	20352	359732	339.381	15659	0.022	0.999	0.001
	Linkage Group LG34	32178	466365	434.188	5688	0.076	0.999	0.001
	Linkage Group LGE22	130026	478974	348.949	4399	0.079	0.997	0.003

suavis Past (SKIN 447773)	unlocalised scaffold	472270	1222197	749.928	5548	0.135	0.997	0.003
hypolizus Present (L62)	1	442962	910758	467.797	17885	0.026	0.999	0.001
	1A	135786	547793	412.008	13671	0.03	0.999	0.001
	1A	74451987	74871868	419.882	8151	0.052	0.998	0.002
	2	129513391	129813597	300.207	13663	0.022	0.999	0.001
	4A	20756223	21058889	302.667	12752	0.024	0.999	0.001
	5	4858	398024	393.167	21450	0.018	0.999	0.001
	21	6344215	6793021	448.807	14928	0.03	0.999	0.001
	25	442111	995671	553.561	8610	0.064	0.998	0.002
	25	995672	1511437	515.766	11073	0.047	0.999	0.001
	27	4343016	4737943	394.928	9375	0.042	0.998	0.002
	28	3899087	4358582	459.496	10948	0.042	0.999	0.001
	Z	55306014	55680829	374.816	17111	0.022	0.999	0.001
	unlocalised scaffold	1011	323995	322.985	22208	0.015	0.999	0.001
	unlocalised scaffold	137	328504	328.368	16335	0.02	0.999	0.001
	Linkage Group LG34	48258	466385	418.128	4028	0.104	1	0
	Linkage Group LGE22	18730	327200	308.471	8161	0.038	0.998	0.002
	Linkage Group LGE22	327200	729881	402.682	11740	0.034	0.999	0.001
hypolizus Past (A129560)	21	6087877	6480931	393.055	11362	0.035	0.999	0.001
	25	543055	853655	310.601	9732	0.032	0.998	0.002
	25	853656	1306006	452.351	8698	0.052	0.998	0.002
	unlocalised scaffold	321264	1253057	931.794	10827	0.086	0.999	0.001

	Linkage Group LG34	16223	462324	446.102	10824	0.041	0.999	0.001
	Linkage Group LGE22	166013	582463	416.451	8498	0.049	0.998	0.002
melanurus Present (L86)	1	544108	856512	312.405	8200	0.038	0.998	0.002
	1A	74415760	74899102	483.343	13476	0.036	0.999	0.001
	4A	20756269	21063916	307.648	14746	0.021	0.999	0.001
	21	5994908	6300537	305.63	10090	0.03	0.999	0.001
	21	6549000	6908600	359.601	17904	0.02	0.999	0.001
	25	436856	921939	485.084	7359	0.066	0.998	0.002
	25	921940	1347448	425.509	3931	0.108	0.996	0.004
	27	4135455	4704985	569.531	12043	0.047	0.999	0.001
	Z	53409471	53845167	435.697	16156	0.027	0.999	0.001
	unlocalised scaffold	3176	317234	314.059	20519	0.015	0.999	0.001
	Linkage Group LG34	36389	466626	430.238	5249	0.082	0.998	0.002
	Linkage Group LGE22	118031	593574	475.544	6973	0.068	0.998	0.002
	unlocalised scaffold	221508	628736	407.229	16851	0.024	0.999	0.001
melanurus Past (A129554)	unlocalised scaffold	269455	1232874	963.42	11255	0.086	0.999	0.001
	Linkage Group LG34	16230	436892	420.663	12165	0.035	0.999	0.001

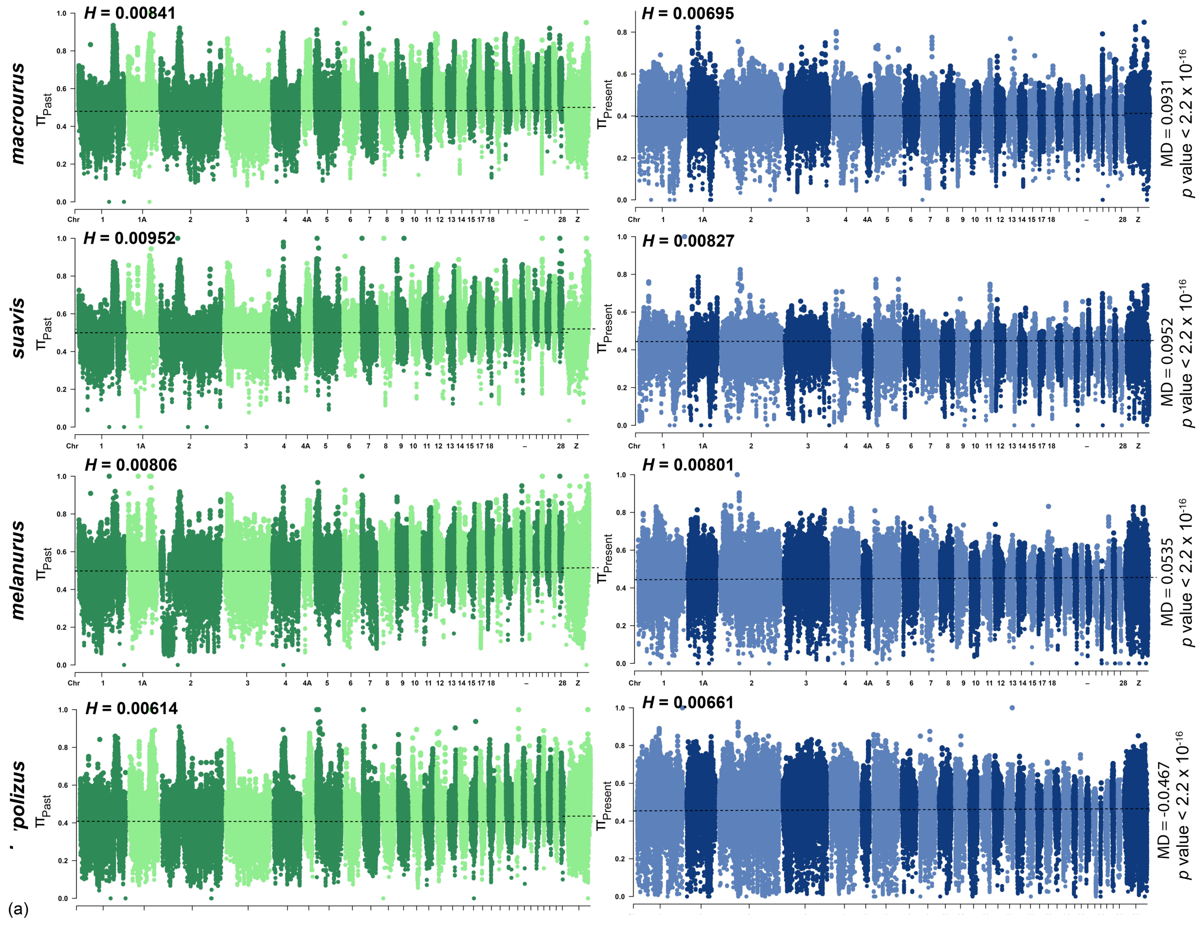

Fig. S8. Comparison of population parameters between two time points across four white-rumped shama taxa – *macrourus, suavis, melanurus, hypolizus* – in the form of Manhattan plots of nucleotide diversity in 50kb sliding windows with a step size of 10kb across the genome for samples collected one century ago (left, green) and modern samples (right, blue). Alternating hues denote different chromosomes. The horizontal black dotted lines denote the mean nucleotide diversity of autosomes and the Z chromosome, which is generally higher in samples collected one century ago than in modern samples.

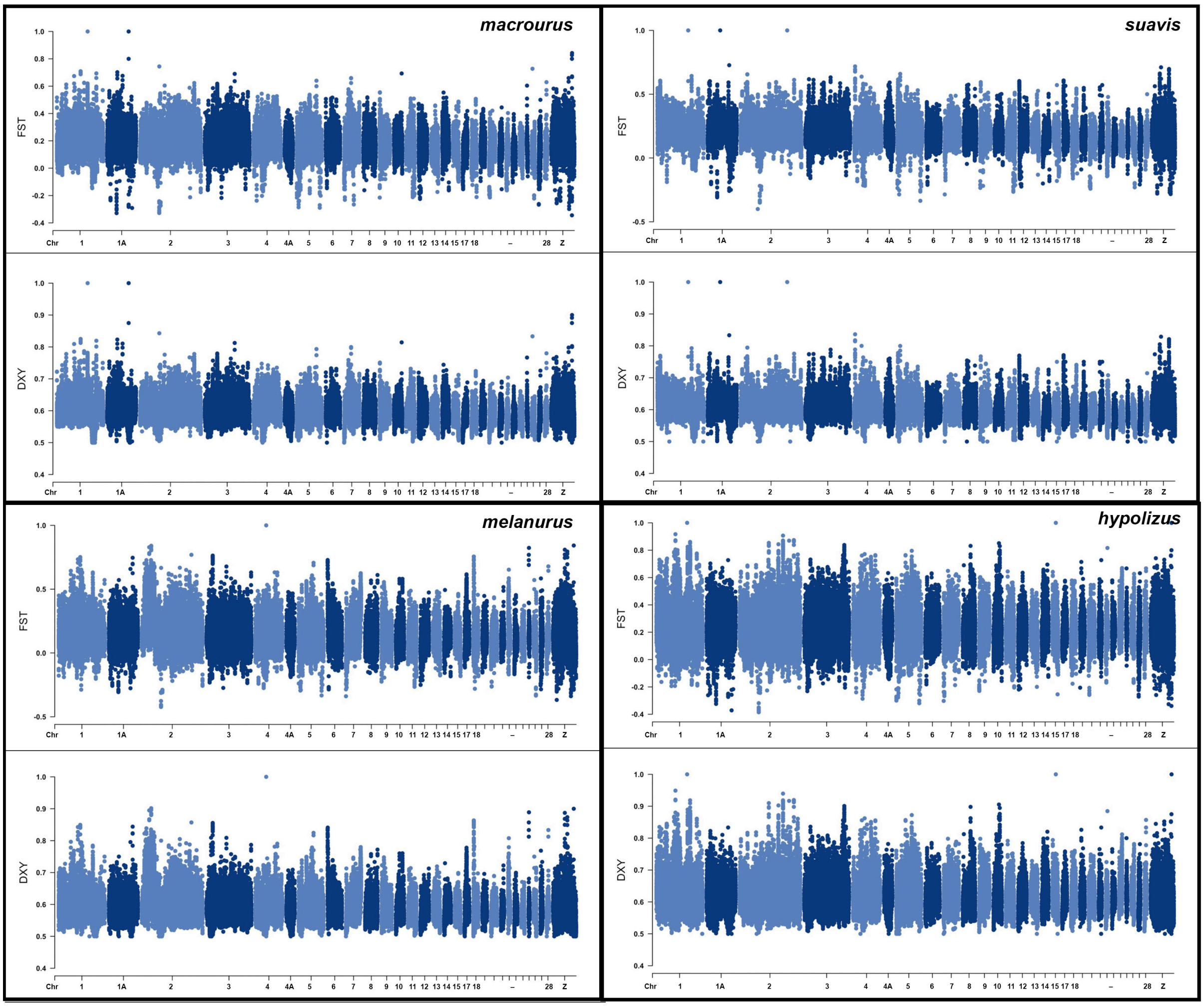

Fig. S9. F_{ST} (top) and D_{XY} (bottom) pairwise divergences between historic and modern white-rumped shama individuals of four taxa (*macrourus, suavis, melanurus, hypolizus*) in 50kb sliding windows with a step size of 10kb across the genome. Alternating hues of blue denote different chromosomes.

Table S9. Basic quality statistics generated by Qualimap for each sample mapped to the Seychelles magpie-robin genome.

**Specimen ID/Voucher number**	**Number of reads**	**Mapped paired reads (%)**	**Mean coverage**
ZRC 3.22575	42,952,149	100	3.2188
ZRC 3.22599	2,647,164	100	0.18
ZRC 3.22474	8,953,862	100	0.1858
ZRC 3.22482	43,705,277	100	3.0362

ZRC 3.4175	27,507,732	100	1.6546
ZRC 3.22478	17,713,612	100	1.0231
ZRC 3.22515	12,426,693	100	0.3145
ZRC 3.22511	33,688,211	100	0.6434
ZRC 3.22594	22,650,902	100	0.5006
SKIN 580290	44,133,809	100	4.3928
SKIN 580291	3,528,440	100	0.299
SKIN 580388	54,543,733	100	5.0785
SKIN 462930	100,655,940	100	9.8626
SKIN 291682	94,200,346	100	8.1113
SKIN 447772	32,163,106	100	1.2988
SKIN 447773	64,821,596	100	6.3033
SKIN 580368	57,094,266	100	5.0636
SKIN 344447	113,827,479	100	10.1524
RMNH.AVES.129477	82,240,082	100	7.9124
RMNH.AVES.129560	56,230,978	100	6.0954
RMNH.AVES.129549	47,240,517	100	5.1974
RMNH.AVES.129554	83,578,911	100	9.4938
SEA015	35,386,519	100	4.9306
NRM 20036774	35,035,581	100	4.8592
NRM 20036776	37,524,021	100	5.1663
MNHN 33-6D (JF163)	32,064,716	100	4.4545
MNHN 33-05F (JF200)	39,296,515	100	5.4413
MNHN JF318	24,595,752	100	3.4054
MNHN JF401 (Voucher no. CG 2013-194)	47,496,570	100	6.5528
MNHN JF409 (Voucher no. CG 2013-196)	42,673,020	100	5.8771
MNHN JF411	43,602,837	100	6.0663
MNHN JF1793	36,650,641	100	5.0895
MNHN JF1795	40,141,280	100	5.5503
MNHN JF4245	57,516,144	100	7.9374
MNHN JF4287	42,468,542	100	5.8987
MNHN 05-37	59,085,488	100	8.199

MNHN 04-8F	61,016,289	100	8.1295
MNHN 06-106 (MNHN CG 2000-0337)	38,013,741	100	5.1085
KK03	37,621,950	100	5.2427
KK11	39,830,592	100	5.5101
DOT 15021	31,536,897	100	4.3093
DOT 334	45,935,214	100	6.3263
SIM39	44,350,597	100	6.0844
SIM43	49,556,803	100	6.8596
SIM44	46,185,161	100	6.3324
SIM45	47,117,301	100	6.4846
SIM46	40,581,104	100	5.5831
SIM47	40,673,952	100	5.5877
NIA01	41,502,615	100	5.6842
NIA02	44,330,470	100	6.0894
NIA75	47,210,732	100	6.4959
SIB35	54,395,737	100	7.4863
PBI-MAL-052	45,138,785	100	6.1461
PBI-MAL-033	37,316,524	100	5.1072
L1739	30,537,484	100	4.2469
L2328	33,780,298	100	4.7062
L1547	57,892,888	100	7.9361
L2229	44,563,302	100	6.0793
L2241	44,958,615	100	6.038
JBPC2740	57,569,616	100	7.8399

References

Burner RC, Shakya SB, Haryoko T, Irham M, Prawiradilaga DM, Sheldon FH. 2018. Ornithological Observations from Maratua and Bawean Islands, Indonesia. *Treubia* 45:11–24.

Cavill EL, Gopalakrishnan S, Puetz LC, Ribeiro ÂM, Mak SS, Da Fonseca RR, Pacheco G, Dunlop B, Accouche W, Shah N, Zora A. 2022. Conservation genomics of the endangered Seychelles Magpie-Robin (*Copsychus sechellarum*): a unique insight into the history of a precious endemic bird. *Ibis* 164(2):396–410.

Chan CH, Robertson HA, Saul EK, Nia LV, Luong VP, Kong X, Zhao Y, Chambers GK. 2011. Genetic variation in the kakerori (*Pomarea dimidiata*), an endangered endemic bird successfully recovering in the Cook Islands. *Conservation Genetics* 12(2):441–447.

Chua VL, Phillipps Q, Lim CH, Taylor SS, Gawin DF, Rahman MA, . . . Sheldon FH. 2015. Phylogeography of three endemic birds of Maratua Island, a potential archive of Bornean biogeography. *Raffles Bulletin of Zoology* 63:259–269.

Clements JF, Schulenberg TS, Iliff MJ, Billerman SM, Fredericks TA, Sullivan BL, Wood CL. 2019. The eBird/Clements Checklist of Birds of the World: v2019. Retrieved from https://www.birds.cornell.edu/clementschecklist/download/ (accessed 19 September 2020).

Dussex N, Van Der Valk T, Morales HE, Wheat CW, Díez-del-Molino D, Von Seth J, Foster Y, Kutschera VE, Guschanski K, Rhie A, Phillippy AM. 2021. Population genomics of the critically endangered kākāpō. *Cell Genomics* 1(1):100002.

Eaton J, van Balen B, Brickle N, Rheindt FE. 2016. Birds of the Indonesian Archipelago: Greater Sundas and Wallacea. Barcelona, Spain: Lynx Edicions.

Eaton J, van Balen B, Brickle N, Rheindt F. 2021. Birds of the Indonesian Archipelago: Greater Sundas and Wallacea (2nd ed.). Barcelona, Spain: Lynx Edicions.

Forsdick NJ, Cubrinovska I, Massaro M, Hale ML. 2017. Genetic diversity and population differentiation within and between island populations of two sympatric *Petroica* robins, the Chatham Island black robin and tomtit. *Conservation Genetics* 18(2):275–285.

Gill F, Donsker D, Rasmussen P. 2021. IOC World Bird List (v11.1). Retrieved from http://www.worldbirdnames.org/new/ioc-lists/master-list-2/ (accessed 19 September 2020). http://dx.doi.org/10.14344/IOC.ML.11.1

Handbook of the Birds of the World & BirdLife International. 2019. Handbook of the Birds of the World and BirdLife International digital checklist of the birds of the world. Version 4. Retrieved from http://datazone.birdlife.org/userfiles/file/Species/Taxonomy/HBW-BirdLife_Checklist_v4_Dec19.zip (accessed 19 September 2020).

Hebert PDN, Stoeckle MY, Zemlak TS, Francis CM, Godfray C. 2004. Identification of birds through DNA barcodes. *PLoS Biology* 2(10):e312.

Kerr KC, Stoeckle MY, Dove, CJ, Weigt LA, Francis CM, Hebert PD. 2007. Comprehensive DNA barcode coverage of North American birds. *Molecular Ecology Notes* 7(4):535–543.

Li S, Li BO, Cheng C, Xiong Z, Liu Q, Lai J, Carey HV, Zhang Q, Zheng H, Wei S, Zhang H. 2014. Genomic signatures of near-extinction and rebirth of the crested ibis and other endangered bird species. *Genome Biology* 15(12):1–7.

Lim HC, Gawin DF, Shakya SB, Harvey MG, Rahman MA, Sheldon FH. 2017. Sundaland's east–west rain forest population structure: variable manifestations in four polytypic bird species examined using RAD-Seq and plumage analyses. *Journal of Biogeography* 44(10):2259–2271.

Mayr E. 1968. Comments on 'Theories and Hypotheses in Biology.'. Cambridge, United States: Belknap Press of Harvard.

Mayr E. 1992. Species Concepts and their Application. Cambridge, United States: MIT Press.

Ogata M, Ishii H, Onuma M. 2020. Characterization of eight polymorphic microsatellite DNA markers and mitochondrial cytochrome c oxidase subunit II gene in Bali myna *Leucopsar rothschildi*. *International Zoo Yearbook* 54(1):165–173.

Peters JL. 1964. Check-List of the Birds of the World. Vol. 10. Harvard University Press, Cambridge, pp. 69–72. http://dx.doi.org/10.5962/bhl.title.14581

Prasad A, Lorenzen ED, Westbury MV. 2022. Evaluating the role of reference-genome phylogenetic distance on evolutionary inference. *Molecular Ecology Resources* 22(1):45–55.

Rheindt FE, Baveja P, Ferasyi TR, Nurza A, Rosa TS, Haminuddin, . . . Gwee CY. 2019. The extinction-in-progress in the wild of the Barusan Shama *Copsychus* (*malabaricus*) *melanurus*. *Forktail* 35:28–35.

Vorderman AG. 1893. Bijdrage tot de kennis der vogels van den Kangean-archipel. In Koninklijke Natuurkundige Vereeniging in Nederlandsch Indië., Natuurkundig

tijdschrift voor Nederlandsch Indië (Vol. 53). Batavia:Lange.
https://www.biodiversitylibrary.org/bibliography/13350

Voris HK. 2000. Maps of Pleistocene sea levels in Southeast Asia: shorelines, river systems and time durations. *Journal of Biogeography* 27(5):1153–1167.

Wu MY, Rheindt FE. 2022. A distinct new subspecies of the White-rumped Shama *Copsychus malabaricus* at imminent risk of extinction. *Journal of Ornithology* in press.
