# Supplementary material for: Genomes From Historic DNA Unveil Massive Hidden Extinction and Terminal Endangerment in a Tropical Asian Songbird Radiation
Source: Mol Biol Evol. 2022 Sep 6;39(9):msac189. doi: 10.1093/molbev/msac189 (PMC9486911; doi:10.1093/molbev/msac189)
Supplement: msac189_Supplementary_Data [file ResponseLetter.pdf › ResponseLetter.pdf]

<u>Response Letter</u>

Dear Board of Editors,

Thank you very much for the positive response to our revised manuscript. We have carried out all the additional minor changes required. In the following, we will reply to each and every one of the editor's and reviewers' comments to outline the actions we have taken in response to their suggestions and criticisms. Our responses follow each comment and are printed in bold for improved visualization.

<u>Editors' Comments:</u>

We have received two responses to the revisions for your manuscript on songbirds. Both reviewers are happy with the additional analyses. There are a few minor changes to the text and figures that will need to be tidied up. The authors will also need to provide SRA numbers before final acceptance. We appreciate you sending your work to MBE.

>>> We thank the editor for the positive response to our revised manuscript. We have uploaded all files under the accession number PRJNA867483 on SRA.

<u>Comments of Reviewer 1:</u>

I think the authors have done a great job of dealing with reviewer's comments and I look forward to seeing the paper published soon.

>>> We thank the reviewer for acknowledging our efforts in improving our manuscript.

<u>Comments of Reviewer 2:</u>

The authors have added a number of useful analyses and visualizations. I have a few minor comments that should be able to be addressed without significant additional computation.

1. It is still less clear that it should be exactly what features of the data the authors are using to determine proposed species splits. I am not a systematist (neither is I suspect the median reader of MBE), but to me it is somewhat hard to see why, for example, leggei and stricklandii are proposed as a separate species, but suavis is not (I am not sure why the logic that suavis appears distinct because of gene flow doesn't also apply to stricklandii). Really, it is not entirely clear to me on what basis any species besides the omissus/javanus clade is split from the mainland clade. However, I don't want to belabor the point. I think the additional analyses the authors have added strengthen the basic result that a lot of hidden diversity exists in the island edges of the species complex range, and I don't want to quibble too much about a specific proposal for species splits, especially as it may in part be motivated by conservation needs. So some small discussion, or expanding a little bit the text in the supplement for curious readers, would probably be enough to resolve this.

>>> We have added substantial detail to our taxonomic revision in the Supplement, clarifying the species concept and criteria applied, and providing considerable additional level of morphological and genomic detail on what has guided our taxonomic recommendations. We even added discussions on Earth history and land connections that would have enabled recent gene flow among now-insular taxa, furnishing the background for why some of the distinct phylogenetic positions were so surprising and demanded elevation of taxa to species level. In our previous manuscript version, we did not go into such detail as we were afraid it may provide too much taxonomic discussion in what's meant to be a non-taxonomic paper. However, we fully agree with the reviewer that such detail is laudable and necessary in the Supplement, helping readers follow

our taxonomic rationale. Hence, we were glad to update the taxonomic revision part accordingly. We hope the additional information satisfies the reviewer's concerns.

2. The admixture plot is quite difficult to interpret, and certainly does not seem to show strong evidence for clear, distinct, isolated populations. Part of the issue I believe is that the colors are inconsistent and it can be hard to see which groups go with which (e.g., there are light blue chunks at both the far left and middle right parts of the plot for K=7, and this is common). The program pong (https://ddec1-0-en-ctp.trendmicro.com:443/wis/clicktime/v1/query?url=https%3a%2f%2fgithub.com%2framachandran%2dlab%2fpong&umid=bacbd607-6e83-4474-b56d-918db368b28c&auth=8d3ccd473d52f326e51c0f75cb32c9541898e5d5-572106389fcc622f14ff60d80188d656cfbcf69d) can help make multi-K structure plots like this easier to interpret and display, and I would recommend improving this plot before publication. It would also be useful to know the best-fit K.
>>> We appreciate the reviewer's suggestion to use pong for better illustration of our NGSadmix results. We have exchanged the plot to display the one from pong in the supplement, and we have added the best *K* value to the caption.

3. Although I take the author's point that the species tree is poorly resolved, the SNAPPER paper analyzes a turtle species complex that seems to have similar divergence as the species studied here, and uses much more interpretable plots (e.g., Fig 5 and Fig 6, here https://ddec1-0-en-ctp.trendmicro.com:443/wis/clicktime/v1/query?url=https%3a%2f%2farxiv.org%2fpdf%2f1909.07276.pdf&umid=bacbd607-6e83-4474-b56d-918db368b28c&auth=8d3ccd473d52f326e51c0f75cb32c9541898e5d5-faf8a848dd431476bb6bf5df84d403ac6657b164). I think this would be a much clearer way to display the results of this analysis, if it is possible generate from the authors data.
>>> We thank the reviewer for this suggestion. However, we were unable to change the display of our results. The program used for cloudogram visualisation as requested by the reviewer is DensiTree, which does not allow us to reroot the tree to the outgroup. SNAPPER also does not allow for analyses with a rooted tree. When using these programs without rooting, they produce erroneously rooted topologies. The problem with rooting most likely stems from the poor resolution of the tree and we do not wish to showcase a tree with an erroneously rooted topology. We hope the reviewer understands our point of view.

4. I think one of the PCA or the admixture plot (after cleanup and improvement) would be worth including in the main text, probably along with some basic population differentiation stats such as Fst. The authors only have two figures right now, and some figure that shows an overall summary of the within and between species diversity would be a useful addition.
>>> We thank the reviewer for this suggestion. We have added the PCA as our new fig. 2 visualising four principal components. We have also calculated and included global population summary statistics (π, F_{ST}, D_{XY}) into the figure to showcase the within and between taxon diversity of our complex.
